# Comparison of gastric reactance with commonly used perfusion markers in a swine hypovolemic shock model

**DOI:** 10.1186/s40635-022-00476-1

**Published:** 2022-11-18

**Authors:** María M. Godinez-Garcia, Adrian Soto-Mota, Jorge Catrip, Ruben Gaitan, Ma del C. Lespron, Francisco J. Molina, Miguel A. Falcón, Alberto Aranda, Carlos A. Tena, Pedro Zamudio, Ivan Briseño, Rolando Alvarez, Yazmin Guillen

**Affiliations:** 1 Instituto Nacional de Cardiología “Ignacio Chavez” (Spanish Acronym INCICH), Mexico City, Mexico; 2 Instituto Nacional de Enfermedades Respiratorias “Ismael Cossio Villegas” (Spanish Acronym INER), Mexico City, Mexico; 3 Unidad de Investigación en Enfermedades Metabólicas, Instituto Nacional de Ciencias Médicas y Nutrición “Salvador Zubirán” (Spanish Acronym UIEM-INCMNSZ)”, Mexico City, Mexico; 4 Alandra Medical SAPI de CV, Mexico City, Mexico

**Keywords:** Electrical Impedance, Hypovolemic shock, Gastric reactance, Hypoperfusion

## Abstract

**Background:**

The gut has been hypothesized to be a protagonist tissue in multiple organ dysfunction syndrome (MODS) for the past three decades. Gastric reactance (XL) is a potential perfusion marker derived from gastric impedance spectroscopy (GIS), which is an emerging tool through which living tissue can be continuously measured to determine its pathophysiological evolution. This study aimed to compare the performance of XL [positive predictive values (PPV), negative predictive values (NPV), and area under the curve (AUC)] against commonly used perfusion markers before and during hypovolemic shock in swine subjects.

**Methods:**

Prospective, controlled animal trial with two groups, control group (CG) *N* = 5 and shock (MAP ≤ 48 mmHg) group (SG) *N* = 16. Comparison time points were defined as T-2 (2 h before shock), T-1 (1 h before shock), T0 (shock), T1 (1 h after shock), and T2 (2 h after shock). Shock severity was assessed through blood gases, systemic and hemodynamic variables, and via histological examination for assessing inflammation-edema and detachment in the gastric mucosa. Macroscopic assessment of the gastric mucosa was defined in five levels (0—normal mucosa, 1—stippling or epithelial hemorrhage, 2—pale mucosa, 3—violet mucosa, and 4—marmoreal mucosa). Receiver Operating Characteristic (ROC) curves of perfusion markers and XL were calculated to identify optimal cutoff values and their individual ability to predict hypovolemic shock.

**Results:**

Comparison among the CG and the SG showed statistically significant differences in XL measurements at T-1, T0, T1, and T2, while lactate showed statistically significant differences until T1 and T2. Statistically significant differences were detected in mucosa class (*p* < 0.001) and in inflammation-edema in the gastric body and the fundus (*p* = 0.021 and *p* = 0.043). The performance of the minimum XL value per subject  per event (XL_Min) was better (0.81 ≤ AUC ≤ 0.96, 0.93 ≤ PPV ≤ 1.00, 0.45 ≤ NPV ≤ 0.83) than maximum lactate value (Lac_Max) per subject per event (0.29 ≤ AUC ≤ 0.82, 0.82 ≤ PPV ≤ 0.91, 0.24 ≤ NPV ≤ 0.82). Cutoff values for XL_Min show progressive increases at each time point, while cutoff values for Lac_Max increase only at T2.

**Conclusions:**

XL proved to be an indirect and consistent marker of inadequate gastric mucosal perfusion, which shows significant and detectable changes before commonly used markers of global perfusion under the hypovolemic shock conditions outlined in this work.

**Supplementary Information:**

The online version contains supplementary material available at 10.1186/s40635-022-00476-1.

## Take home message

XL could be a clinically useful marker for early detection of hypovolemic shock.

## Introduction

Timely recognition of the deterioration of tissue perfusion is a primordial objective while treating patients with hemodynamic instability, as failure in conducting prompt interventions to restore effective perfusion often results in organ dysfunction and death. Notwithstanding its clinical relevance, identifying early signs of hypoperfusion remains a fundamental problem in perioperative and critical care, because most standard practice resources (such as mixed venous oxygen saturation (SvO_2_), carbon dioxide (CO_2_) gap, lactatemia, and capillary refill time) frequently fail to identify the early signs of end-organ damage [1].

Throughout the past three decades, technological innovations have targeted "canary organs", which can be easily and safely accessed and whose perfusion is believed to deteriorate before other organ beds, therefore, providing an early warning of systemic hypoperfusion. Pioneering technologies include gastric tonometry [2], sublingual capnometry [3], and microcirculation imaging [4], whereas recent developments include gastric impedance spectroscopy (GIS) [5–12], urethral photoplethysmography [13], and bladder tissue oxygen monitoring [14]. However, gastric tonometry fell out of favor, [15] and sublingual capnometry devices are no longer commercially available [16], while microcirculation imaging has acquired increasing relevance in intensive care [4]. On the other hand, GIS, urethral photoplethysmography, and bladder tissue oxygen monitoring hold promise to provide meaningful information to improve patient outcomes.

This study is concerned with GIS, a technique that surveys passive electrical properties of the gastric wall to provide a continuous marker called *gastric reactance* (XL). The XL marker has been proposed as an early warning of multiple organ dysfunction, as it responds to the overall hypoxic status of gastric tissue, which is affected by persistent changes in local and systemic perfusion [5, 7, 10].

XL is measured through a feeding tube provided with an impedance sensor controlled by a bedside monitor that displays instantaneous and trend values of this marker. This configuration is advantageous as feeding tubes are routinely used in critical care settings, and no additional catheterization is needed to keep track of this surrogate perfusion marker.

This study aimed to compare the performance of XL against the performance of commonly used perfusion markers before and during hypovolemic shock in swine subjects divided into two groups: a control group (CG) and a shock group (SG), where shock was defined as MAP ≤ 48 mmHg. A swine hemorrhage model was chosen, since cardiovascular and hemodynamic responses [17, 18], as well as gastrointestinal physiological properties [19], are comparable to a human. The variables selected for this study were based on internationally accepted clinical practice.

## Methods

### Ethical statement

The study was approved by the Committee on Animal Experimentation at the National Institute of Cardiology—Ignacio Chavez (INCICH) in Mexico City (reference number 09F12). Anesthesia was used in all surgical interventions. Experiments described in this study were performed in adherence to Good Laboratory Practices (GLP) [20, 21], National regulations NOM-062-ZOO-1999 [22], General Considerations for Animal Studies for Cardiovascular Devices [23], and Animal Research: Reporting of In Vivo Experiments (ARRIVE guidelines) [24] (Additional file 1: Table S1). The study was monitored by an independent quality assurance unit.

### Sample size

Previous experimental protocols in an ischemia–reperfusion swine model have determined a standard deviation for XL of 0.20 − *j*Ω [25, 26], considering a significance with a *p* value < 0.05, a confidence interval of 0.95, and a margin of error of 10%, the sample size for the shock group is at least 15 subjects, and 6 subjects for the control group.

### Experimental protocol

**Fig. 1:**
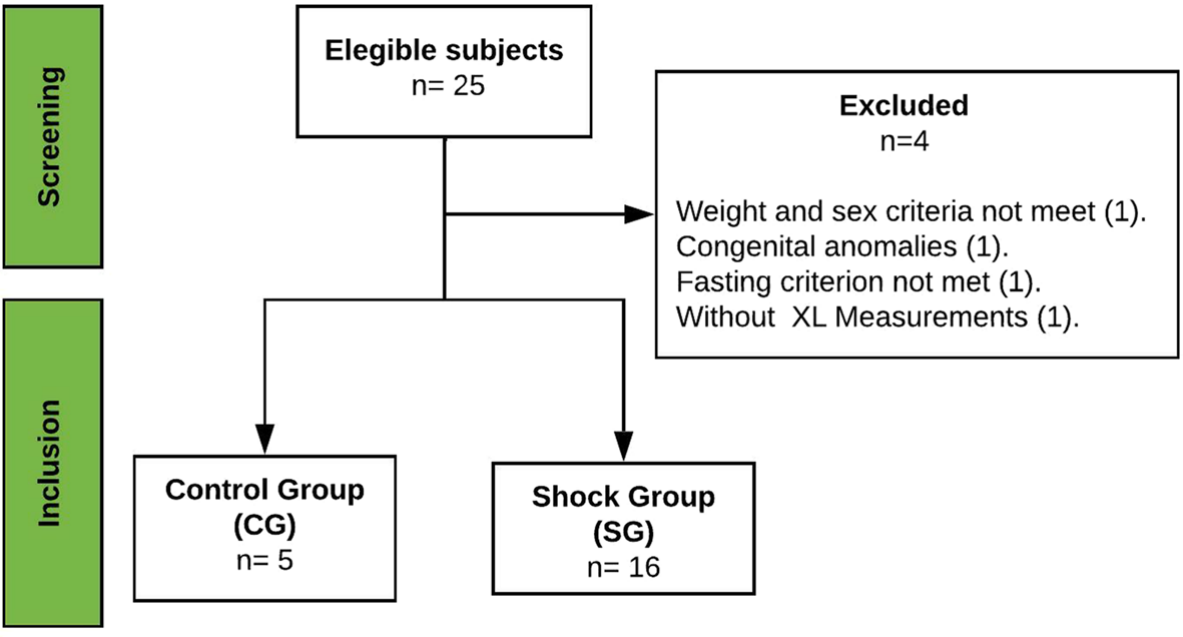
Subject’s flow chart

The study screened 25 male York-Landrace swine subjects sourced from a licensed vendor (Centre for Education, Research and Extension in Pig Production, Mexico). Four subjects were excluded for not meeting inclusion criteria (health certificate, 27–35 kg, castrated, fasting ≥ 16 h), and only 21 subjects were included (Fig. 1). The study was divided into two stages, in the first stage, baseline measurements of gastric reactance, vital signs, laboratory tests, and histopathological samples were taken from the CG of 5 subjects. In the second stage, the same measurements plus hemodynamic variables were taken from the SG of 16 subjects; in this case, baseline conditions were followed by induced hypovolemic shock with a controlled hemorrhage at a rate of 15–20 mL/min until a MAP of 40–30 mmHg was achieved (Additional file 2: Fig. S1).

#### Housing and husbandry

All swine were transported safely with proper ventilation, and the target temperature ranges were defined as 10–29 °C. A veterinarian performed the clinical examination, codification, and placed each subject in a barnyard. The swine were acclimatized to the environment under a regular diurnal cycle (12:12 light:dark) with ad libitum access to water and a controlled food intake, chopped corn cob was used as bedding, and vital signs were recorded daily until the procedure day. In addition, all animals underwent fasting for at least 16 h before surgery but were allowed water ad libitum.

#### Anesthesia

All subjects were pre-treated with atropine (0.1 mg/kg, intramuscular), Zoletil^®^ (tiletamine hydrochloride and zolazepam hydrochloride [7–10 mg/kg, intramuscular]), and azaperone (4 mg/kg, intramuscular). Continuous inhaled isoflurane (1–2%) was used to maintain anesthesia during the experiment, and the rate was adjusted as needed to provide adequate anesthesia; all variations from this rate were recorded (Vaporizer, Datex Ohmeda Tec 4).

#### Tracheostomy and mechanical ventilation

A midline cervical incision was made, and a tracheostomy was performed to warrant adequate ventilation (Modulus II plus, Datex Ohmeda). Initial settings were tidal volume (VT) 12 (mL/kg), respiratory rate (RR) of 12–14/min titrated to maintain PaCO_2_ within the normal range (35–45 mmHg), fraction of inspired oxygen (FiO_2_) of 75%, inspiration/exhalation ratio 1:2, and positive end-expiratory pressure (PEEP) of 3 cmH_2_O.

#### Injury

Hemorrhage in the SG was initiated after a period of 20–40 min baseline stabilization. Continuous gravity exsanguination was performed at a target rate of 15–20 mL/min until reaching a mean arterial pressure (MAP) of 30–40 mmHg, with 40% of the total blood volume (TBV) withdrawn. Total blood volume was estimated from each subject’s weight in kg (W) according to the formula TBV = 65 mL/kg*W (mL) determined by [27]. Volume was continuously recorded, and blood was refrigerated for the duration of the experiment. Hypovolemia and hypotension conditions were maintained for about 4 h by infusing and titrating crystalloids according to the requirements of the subject. No vasopressors were administered to the subjects.

#### Shock criterion

The shock criterion was established as MAP ≤ 48 mmHg, which is two standard deviations below the mean MAP of the CG. Considering this, the relevant time points to be evaluated are T-2: 2 h before shock; T-1: 1 h before shock; T0: shock; T1: 1 h after shock; T2: 2 h after shock.

#### Euthanization

After all study procedures were completed, an intravenous injection of potassium chloride solution 1–2 mmol/kg was given to induce euthanasia.

### Surgical preparation

After subjects were prepped and draped in a sterile fashion, a left carotid artery catheter was placed for blood chemistry, blood gas measurements (Radiometer, ABL 835), and systemic arterial pressure monitoring (Matron, BPM-1000). A double-lumen catheter was inserted in the right internal jugular vein for fluid and drug administration. A left external jugular pulmonary artery catheter (PAC) (7.5 French, model 774F75, Edwards Lifesciences) using Medex transducers (Pressure Transducer MX9504, Medex) was placed for measuring hemodynamic variables, cardiac output, and sampling mixed venous blood gases (Edwards, Monitor Vigilance II), and a Foley catheter was also placed. In both groups, temperature was regulated through either an electric blanket to avoid hypothermia or through ice bags when subjects drifted towards hyperthermia (rectal measurements > 38 °C).

### Measurements

#### Vital signs and hemodynamic

Vital signs such as electrocardiogram, pulse oximetry, temperature, mean arterial pressure (MAP), heart rate (HR), and central venous pressure (CVP) were measured every 30 min in both groups. Hemodynamic variables acquired through the PAC (only from the SG) were: SvO_2_, pulmonary artery systolic pressure (PASP), pulmonary artery diastolic pressure (PADP), pulmonary artery wedge pressure (PAWP), cardiac output (CO), systemic vascular resistance (SVR), pulmonary vascular resistance (PVR), and stroke volume (SV) among others. Calculated variables such as oxygen delivery (DO_2_), oxygen consumption (VO_2_), arterial oxygen content (CaO_2_), venous oxygen content (CvO_2_), oxygen extraction ratio (REO_2_), and mean pulmonary artery pressure (MPAP) were determined through the PAC software, that applies the equations described in [28–30]. Additional parameters required to calculate these variables were obtained from blood gas readings.

#### Gastric impedance spectroscopy and gastric reactance (XL)

XL is a marker derived from the electrical impedance of the gastric wall, a feature that provides a distinctive fingerprint of tissue structure; pathologies such as ischemia, infarct, or necrosis cause cellular alterations that are reflected by changes in electrical impedance. The biological and mathematical basis for the calculation of XL has been published in peer-reviewed scientific literature by independent research groups [5, 6, 8, 31]. The GIS device (Additional file 2: Fig. S2) determines the magnitude of XL through proprietary algorithms applied to impedance readings which are acquired through a feeding tube fitted with an impedance sensor.

During the experiment, correct positioning of the feeding tube and the impedance sensor was confirmed endoscopically (Fujinon 200). After this, the tube was connected to the bedside monitor and XL measurements were taken every minute.

#### Endoscopy and histology

For the CG, a set of biopsies from the body, the fundus, and the antrum of the stomach were taken, one set after tracheostomy and one set at the end of the procedure. For the SG, the same sets were taken, in this case, one after tracheostomy, two during hemorrhage, and one at the end of the procedure. Endoscopic assessments of the gastric wall were performed during every biopsy in both groups. All biopsies were stored in a 3.7 M phosphate buffer (formol), pH 7.2 solution, and then embedded in paraffin to be cut with a microtome to a thickness of 4 microns and then stained with hematoxylin–eosin. A microscopic description and a photomicrography were documented for each cut. A qualitative assessment was made for each biopsy identifying superficial inflammation, edema, and superficial detachment by a pathologist following the empirical scale: 0%, 12.5%, 25%, 50%, 75%, and 100%. On the other hand, the macroscopic appearance of the gastric mucosa was classified by a gastroenterologist into five levels: normal mucosa (level 0), stippling or epithelial hemorrhage (level 1), pale mucosa (level 2), violet mucosa (level 3), and marmoreal mucosa (level 4).

#### Laboratory tests

Venous and arterial blood gases were measured in both groups every 30 min (ABL 835 Radiometer Blood gas analyzer). Blood gas samples include: pH, partial pressure of carbon dioxide in arterial blood (PaCO_2_), partial pressure of oxygen in arterial blood (PaO_2_), hemoglobin (Hb), sodium (Na^+^), potassium (K^+^), chloride (Cl^−^), ionized calcium (Ca^2+^), glucose, base excess (ECF), partial pressure of carbon dioxide in venous blood (PvCO_2_), partial pressure of oxygen in mixed venous blood (PvO_2_), carbon dioxide (CO_2_), arterial oxygen saturation (SaO_2_), and lactate.

On the other hand, the following laboratory tests were performed at baseline conditions and at the end of the experiment in both groups: blood creatinine, urea, alanine aminotransferase (ALT), aspartate aminotransferase (AST), total bilirubin, lactate dehydrogenase (LDH), alkaline phosphatase (ALP), fibrinogen (Daytona RX, Randox), prothrombin time (PT), activated partial thromboplastin time (PTT) (Thrombotimer, Behnk Elektronic), complete blood count (CBC) with cell differentials, white blood cell count (WBC), platelet count (BC-2800Vet, Mindray), and complete blood chemistry.

All laboratory tests were carried out at the Clinical Pathology Department of the Faculty of Veterinary Medicine at the National Autonomous University of Mexico.

#### Necropsy

Necropsies were performed on 21 subjects through a thoracoabdominal incision, and all organs in the thoracic and abdominal cavities were collected. Macroscopic photographs of the outer and mucosal surfaces of the stomach were taken. All organs were fixed for 48 h in 10% buffered formalin. Samples from different organs were preserved: fundus, body, and antrum of the stomach, duodenum, ileum, large intestine, lungs, liver, spleen, and kidneys. Tissue samples were embedded in paraffin, cut with a microtome to a thickness of 4 microns, and then stained with hematoxylin–eosin.

### Statistical analysis

Continuous variables were tested for normality using the Shapiro–Wilk test using the residuals method. A non‐parametric one-way ANOVA (Kruskal‐Wallis test) was performed to detect significant differences between the CG and the SG at the relevant time points defined by the shock criterion (all subjects were considered); the main effects were evaluated through post hoc Dunn's multiple‐comparison test to control type I error. One-way repeated measures ANOVA by ranks (Friedman test) were made to detect significant differences between each relevant time point using paired data from the SG (same subjects per time point group); the main effects were evaluated with post hoc Durbin's multiple‐comparison test to control type I error. Holm adjustment method for *p* values for multiple comparisons was used [32]. In all cases, differences were considered significant at a two-tailed *p *value < 0.05.

Receiver operating characteristic (ROC) curves were calculated to compare the ability of lactate and XL to predict hypovolemic shock at each time point, considering the predefined shock criterion (MAP ≤ 48 mmHg) as the binary target variable. As various measurements of lactate and XL were captured on each time window, ROC curves were built considering only the extreme values of these markers at each window. Maximum lactate measurements (Lac_Max) were chosen, as it is generally accepted that soaring lactatemia is a marker of shock. Similarly, soaring XL measurements have been documented to indicate hypoxia and metabolic stress [5–12]; however, minimum values (XL_Min) were chosen for building the curves to consider the worst-case scenario for the XL marker. In other words values were chosen to favor the predictive performance of lactate while undermining the performance of XL. All curves were computed with a 95% confidence interval (CI) applying stratified bootstrapping (2000 replicates) according to the methods described in [33].

Confounders were considered in the design of statistical analysis. All analyses were conducted using the R Statistical language (version 4.0.5) and RStudio (version 1.4.1106) on Windows 10 using the packages pROC (version1.17.0.1) [33], gtsummary (version 1.4.1) [34] and ggstatsplot (version 0.8.0) [35].

## Results

### Animal characteristics and blood loss

Mean weight of the 21 subjects was 29.6 ± 2.5 (mean ± SD) kg, fasting time was 17.6 ± 1.0 (mean ± SD) h, temperature at transportation was 19.40 ± 2.00 (mean ± SD) °C, and mean acclimatization period was 7.0 ± 3.3 (mean ± SD) days. No significant differences were found between groups. The mean total hydric balance was significantly different between groups (*p* < 0.001), for the CG the balance was 1769.6 ± 578.1 (mean ± SD) mL, while the balance for the SG was − 676.1 ± 762.9 (mean ± SD) mL. The mean total amount of blood loss was 916.8 ± 230.2 (mean ± SD) mL for the SG.

### Gastric reactance and physiological variables

Data are presented by groups (T-2, T-1, T0, T1, T2), as subjects reached the shock criterion at different points in time, making timewise comparisons impractical. Instead, the analysis is tied to the physiological status of subjects defined by the shock criterion, which in itself is defined as a significant deviation from baseline conditions (MAP ≤ 48 mmHg, which is two standard deviations below the mean of the CG).

Table 1 and Additional file 3: Table S2, Figs. S3–S24 report XL, blood gases, vital signs, and hemodynamic measurements for the CG and the SG at relevant time points. XL, lactate, HR, PaO_2,_ and temperature show an upward trend in the SG, while PaCO_2_, MAP, CVP, PAWP, CO, and SvO_2_ show a downward trend in the same group. Significant statistical differences are observed between the CG and the SG. As expected, temperature raised above 38° C, hyperthermia was probably secondary to the metabolic response to trauma in T0 (shock state). Heart rate increased significantly in T0, T1, and T2.

**Table 1:** Gastric reactance, blood gases, vital signs, and hemodynamic comparisons for the CG and the SG time points

	CG	SG					
Variable	N = 62	T-2 N = 31	T-1 N = 39	T0 N = 37	T1 N = 36	T2 N = 21	*p*
Gastric reactance							
XL (− *j*Ω)	8.9 [7.1, 11.5]	10.8 [8.2, 14.7]	14.9 [11.5, 20.2]	15.6 [8.2, 21.2]	19.8 [15.4, 33.8]	25.7 [19.7, 38.3]	< 0.001*
Blood gases							
Lactate (mmol/L)	2.1 [1.8, 3.4]	2.0 [1.8, 2.8]	2.3 [1.9, 3.0]	2.9 [2.3, 4.0]	5.8 [3.4, 8.2]	8.9 [5.9, 10.1]	< 0.001*
pH	7.4 [7.4, 7.5]	7.5 [7.4, 7.5]	7.5 [7.4, 7.5]	7.4 [7.4, 7.5]	7.4 [7.4, 7.5]	7.4 [7.3, 7.4]	< 0.001*
PaCO_2_ (mmHg)	38.4 [34.5, 40.9]	36.8 [33.4, 39.5]	35.2 [33.8, 38.5]	36.1 [31.3, 40.6]	32.0 [28.5, 36.1]	32.9 [26.9, 35.1]	< 0.001*
PaO_2_ (mmHg)	179.0 [150.2, 197.8]	166.0 [149.2, 199.0]	180.0 [139.0, 202.0]	193.0 [152.0, 231.0]	210.0 [140.0, 229.5]	220.5 [160.2, 242.8]	0.170
Vital signs							
HR (bpm)	100.0 [92.0, 107.0]	118.0 [101.5, 131.5]	117.0 [95.5, 157.0]	136.0 [101.0, 165.0]	138.0 [100.0, 162.5]	135.0 [120.0, 151.5]	< 0.001*
MAP (mmHg)	69.0 [60.5, 72.0]	59.0 [53.0, 61.5]	51.0 [50.0, 55.0]	39.0 [36.0, 44.0]	32.0 [29.0, 36.5]	33.0 [27.5, 37.5]	< 0.001*
Temperature (°C)	38.0 [37.7, 38.2]	38.1 [37.0, 39.0]	38.7 [37.8, 39.4]	39.0 [38.0, 39.5]	39.1 [38.2, 39.9]	39.4 [39.0, 39.8]	< 0.001*
CVP (mmHg)	10.0 [9.0, 11.0]	8.0 [7.0, 9.0]	8.0 [6.0, 12.0]	7.5 [4.0, 9.2]	6.0 [3.0, 9.0]	7.0 [3.0, 8.2]	< 0.001*
Hemodynamic							
PAWP (mmHg)	–	10.0 [8.0, 15.8]	7.0 [5.0, 14.2]	7.0 [5.0, 12.5]	8.0 [6.0, 11.0]	6.0 [4.0, 8.0]	0.015*
CO (L/min)	–	3.5 [3.3, 4.8]	3.2 [2.6, 3.9]	2.9 [2.3, 3.4]	2.6 [2.2, 2.9]	2.9 [2.2, 3.1]	< 0.001*
SvO_2_ (%)	–	66.0 [48.2, 76.0]	62.0 [49.0, 72.8]	55.0 [49.0, 61.5]	47.0 [33.2, 60.0]	53.0 [47.0, 57.0]	< 0.001*

Data presented as Median [IQR]; *p* for Kruskal–Wallis rank-sum test. *for statistically significant results (*p* < 0.05)

*bpm* beat per minute, *CG* control group, *CO* cardiac output, *CVP* central venous pressure, *HR* heart rate, *MAP* mean arterial pressure, *PaCO*_*2*_ partial pressure of carbon dioxide in arterial blood, *PaO*_*2*_ partial pressure of oxygen in arterial blood, *PAWP* pulmonary artery wedge pressure, *SG* shock group; *SVO*_*2*_ mixed venous saturation, *XL* gastric reactance

Table 2 shows the renal and electrolyte measurements for the CG and the SG. Significant differences are observed in urea, creatinine, K^+^, Na^+^, and Cl^−^. All laboratory parameters are reported in Additional file 4: Table S3.

**Table 2:** Renal and electrolytes panel

Variable	Control group *N *= 5	Pre-shock *N* = 16	Post-shock *N* = 16	*p*
Urea (mmol/L)	2.50 [2.4, 3.65]	2.28 [1.8, 3.09]	4.80 [4.2, 6.50]	< 0.001*
Creatinine (µmol/L)	83.96 [73.2, 92.25]	87.50 [74.8, 95.25]	151.00 [132.0, 181.00]	< 0.001*
K^+^ (mmol/L)	3.81 [3.7, 3.92]	3.40 [3.3, 4.15]	7.50 [6.6, 8.13]	< 0.001*
Na^+^ (mmol/L)	138.00 [138.0, 138.00]	137.00 [135.8, 137.25]	132.00 [131.0, 133.50]	< 0.001*
Cl^−^ (mmol/L)	102.60 [102.0, 106.00]	99.00 [97.0, 100.00]	98.00 [96.5, 99.00]	0.005*

Data presented as Median [IQR]. *p* for Kruskal–Wallis rank-sum test. *for statistically significant results (*p* < 0.05)

*Cl*^*−*^ chlorine, *K*^+^ potassium, *Na*^+^ sodium

**Fig. 2:**
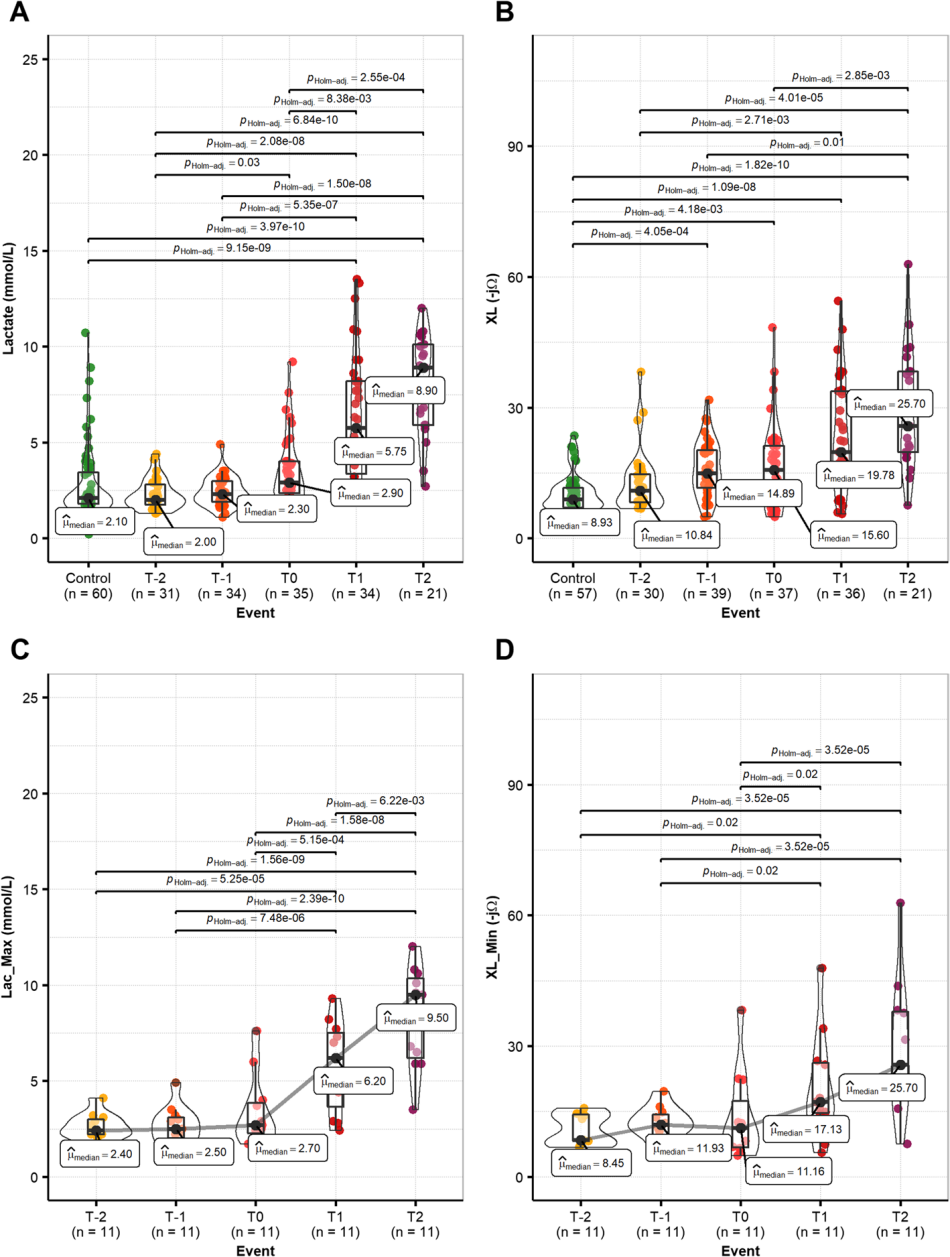
Comparison of Lactate (**A**) and XL (**B**) between the control group (CG) and the shock group (SG). Comparison of the SG for paired data for maximum lactate values per subject per event (Lac_Max) (**C**), and minimum XL values per subject per event (XL_Min) (**D**). Events by shock criterion (MAP ≤ 48 mmHg) T-2: 2 h before shock; T-1: 1 h before shock; T0: shock; T1: 1 h after shock; T2: 2 h after shock. Significant differences between groups are marked as bars with their corresponding *p* values. Post hoc Dunn's all-pairs test for Kruskal–Wallis rank-sum test and post hoc Durbin's all-pairs test for Friedman rank-sum test. Significant *p* values < 0.05. Full Post hoc analysis per variable and time point is available in Additional file 5: Tables S4–S7

On the other hand, Fig. 2A, B and Additional file 5: Tables S4–S5 illustrate the distribution and multiple comparisons of lactate and XL between the CG and the SG at the relevant time points. Both trends behave similarly as they increase as hypovolemic shock progresses.

Nine significant differences in lactate and eight significant differences in XL measurements are observed among the CG and the comparison time points (T-2 to T2) in the SG defined around the shock criterion at T0 (MAP ≤ 48 mmHg); such differences are marked as horizontal bars accompanied with their corresponding *p* value in Fig. 2A, B (bars are only shown for paired data sets presenting significant differences). In the case of lactate, significant differences from the CG measurements are found 1 and 2 h after shock, while in the case of XL, significant differences from the CG measurements are found as early as 1 h before shock and onwards. Other inter-group differences are observed; however, the findings that could be more relevant from the clinical perspective are those related to the earliest identification of hypovolemic shock.

Similarly, Fig. 2C, D and Additional file 5: Tables S6–S7 illustrate the multiple comparisons for paired data (*n* = 11). In this case, shock events were considered for XL minimum values (XL_Min) and the maximum lactate values (Lac_Max) per subject per event; such differences are marked again as horizontal bars accompanied with their corresponding *p* value. In this case, both XL_Min and Lac_Max show the earliest significant difference between T-2 and T1, and onwards.

**Fig. 3:**
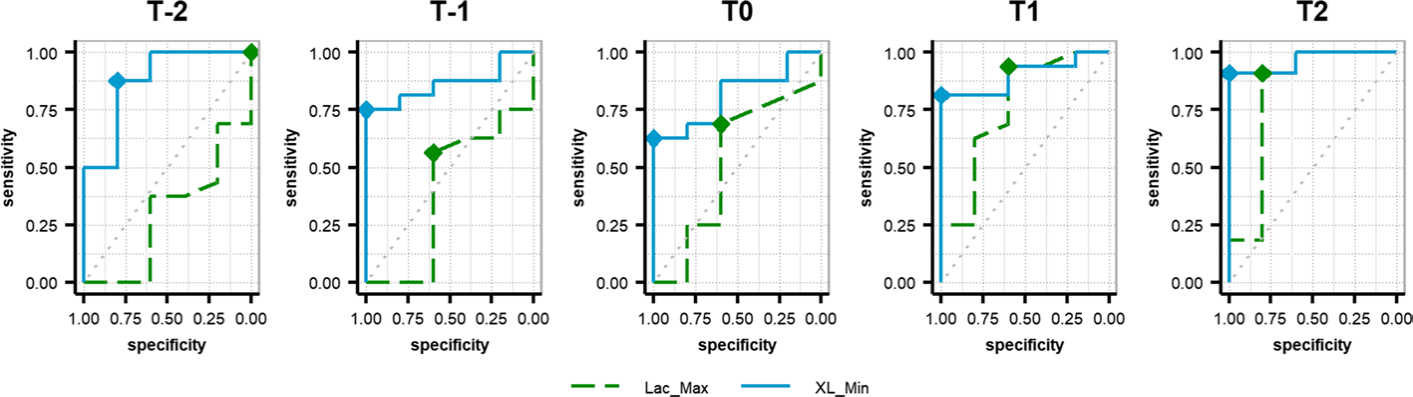
ROC curves for maximum lactate values per subject per event (Lac_Max) and minimum XL values per subject per event (XL_Min) between the control group (CG) and the shock group (SG). All subjects were considered in the relevant time points by the shock criterion (MAP ≤ 48 mmHg). T-2: 2 h before shock; T-1: 1 h before shock; T0: shock; T1: 1 h after shock; T2: 2 h after shock

When comparing the performance of XL_Min and Lac_Max through ROC curves at the relevant time points (T-2, T-1, T0, T1, T2), the area under the curve (AUC), positive predictive value (PPV), and negative predictive value (NPV) is higher for XL_Min (0.81 ≤ AUC ≤ 0.96, 0.93 ≤ PPV ≤ 1.00, 0.45 ≤ NPV ≤ 0.83) than in for Lac_Max (0.29 ≤ AUC ≤ 0.82, 0.82 ≤ PPV ≤ 0.91, 0.24 ≤ NPV ≤ 0.82) at each event (Fig. 3; Table 3).

**Table 3:** Performance for Lac_Max and XL_Min

Time points	*N*	Variable	Optimal cutoff values	Sensitivity	Specificity	AUC	95% CI	PPV	NPV
T-2	16	Lac_Max (mmol/L)	–	0.00	1.0	0.29	0.01–0.58	–	0.24
T-1	16		2.55	0.56	0.6	0.39	0.05–0.74	0.82	0.30
T0	16		2.60	0.69	0.6	0.52	0.17–0.87	0.85	0.38
T1	16		2.65	0.94	0.6	0.76	0.47–1.00	0.88	0.75
T2	11		5.60	0.91	0.8	0.82	0.50–1.00	0.91	0.80
T-2	16	XL_Min (− *j*Ω)	7.79	0.88	0.8	0.88	0.67–1.00	0.93	0.67
T-1	16		9.82	0.75	1.0	0.86	0.70–1.00	1.00	0.56
T0	16		10.18	0.62	1.0	0.81	0.62–1.00	1.00	0.45
T1	16		11.79	0.81	1.0	0.90	0.77–1.00	1.00	0.62
T2	11		12.41	0.91	1.0	0.96	0.88–1.00	1.00	0.83

Performance for maximum lactate values per subject per event (Lac_Max) and minimum XL values per subject per event (XL_Min) between the control group (CG) and the shock group (SG). All subjects were considered in the relevant time points by the shock criterion (MAP ≤ 48 mmHg) T-2: 2 h before shock; T-1: 1 h before shock; T0: shock; T1: 1 h after shock; T2: 2 h after shock

*AUC* area under the curve, *CI* confidence interval, *NPV* negative predictive value, *PPV* positive predictive value

The optimal cutoff values determined by the ROC curves (Table 3) for XL_Min are between 7.79 and 12.41 − *j*Ω, and for Lac_Max are between 2.55 and 5.60 mmol/L. It is observed that XL_Min increases at each time point, while lactate values of 2.55 to 2.65 mmol/L in T-1, T0, and T1 are still within the interquartile range of the CG (Table 1); however, an increase of 3 mmol/L is observed at T2.

### Endoscopy and histology

Endoscopic and histological examinations of the fundus, the body, and the antrum of the stomach were carried out for the CG and the SG. Significant differences in macroscopic mucosa classifications were observed in all three regions (*p* < 0.001). Similarly, significant differences were found in inflammation-edema scores in histologic images from the body and the fundus of the stomach (*p* = 0.021 and *p* = 0.043, respectively). Detailed information in Additional file 6: Tables S8–S14.

## Discussion

In this study, we documented that XL anticipates changes in commonly used perfusion markers. Hemodynamic and metabolic variables confirm that hypovolemic shock was achieved in the intervention group (Tables 1, 2; Additional file 3: Table S2, Figs. S3–S24, Additional file 4: Table S3). Histopathological results show that the gastric tissue remained unaffected in the CG, while gastric tissue in the SG shows signs of inflammation-edema at the body and antrum, and macroscopic assessment of the fundus and the body finds a marble or violaceous appearance, all of which is an indicator of hypoxia at T1 and T2 (Additional file 6: Tables S8–S14).

As expected, the statistical analysis shows that the magnitude of all variables is affected by the reduction of circulating blood volume. In particular, the analysis reveals that significant differences occur earlier in XL than in lactate measurements. Moreover, the analysis reveals that under the conditions in which the experiment was conducted, XL measurements have superior performance for detecting hypovolemic shock: XL_Min (0.81 ≤ AUC ≤ 0.96, 0.93 ≤ PPV ≤ 1.00, 0.45 ≤ NPV ≤ 0.83) vs Lac_Max (0.29 ≤ AUC ≤ 0.82, 0.82 ≤ PPV ≤ 0.91, 0.24 ≤ NPV ≤ 0.82).

These results point in the direction that XL could be a valuable indicator of alterations in systemic perfusion before hypovolemic shock is made evident by other markers. Such timely detection of reduced tissue perfusion would be clinically relevant, as alterations in XL could trigger timely interventions to reduce postoperative complications, avoid multi-organ dysfunction, and improve survival rate in intensive care patients. It should be noted that similar ambitions have been pursued in the past for other markers of gastric perfusion, such as intramucosal pH [36, 37].

Hypotension and hypovolemia during the experiment were persistent enough to trigger major alterations in hemodynamic and metabolic variables, shedding light on the dependency between gastric reactance, perfusion, and metabolic stress. While gastric perfusion was not measured directly, and no conclusions can be drawn about the rate of change of XL in relation to changes in blood flow towards the stomach, results indicate that under these experimental conditions, XL measurements anticipate changes in serum lactate (Fig. 3; Table 3), at the time it shows sensitivity and specificity levels for detecting hypovolemic shock above of those from lactate alone.

On the other hand, it has been reported that XL does not respond to changes in perfusion, even after blood flow towards the superior mesenteric artery is compromised by 25% during hemorrhage and reperfusion maneuvers which were not severe enough to drive relevant changes in tissue lactate [38]. Such observations point in the direction that instead of being sensitive to changes in blood flow, passive electrical properties of tissue are sensitive to structural alterations caused by persistent hypoxia, such as inflammation and edema [39–45].

Our results can only be interpreted in the context of a pilot study intended to inquire about the relationship between XL and commonly used perfusion markers during hypovolemic shock under laboratory conditions. No clear conclusions on the relationship between XL’s rate of change and the duration and severity of hypoperfusion to the stomach can be derived from the experiment. Moreover, the experiment does not address the larger clinical question of whether alterations to mesenteric perfusion announce impending damage to distal organ beds.

In other words, the right cutoffs and operative definitions can make the difference between missing or achieving early and accurate identification of hypovolemic shock. In this work, clear (and potentially useful) differences between XL values were observed before other markers of shock presented significant changes from their baseline (such as lactate and blood pressure).

## Limitations

First, this study was conducted under controlled conditions, which are vastly different from real clinical settings, where the performance of XL could depend on other parameters, such as the patient’s medical history, clinical diagnosis, environment, and conditions of hypovolemic shock, among others. Second, the thresholds found for XL and other variables are only applicable for this breed of swine, and normal values may vary for humans. Third, XL measurements can be distorted by motion artifacts, gastric contents, or incorrect positioning of the impedance sensor at the gastric wall. While defective measurements are easily identifiable through visual inspection of impedance spectra, and signal quality can be retrieved by either draining fluids or moving the catheter, such nuisances could limit the usage of GIS in a real clinical setting.

## Conclusions

XL proved to be an indirect and consistent marker of inadequate gastric mucosal perfusion, which shows significant and detectable changes before commonly used markers of global perfusion under the hypovolemic shock conditions outlined in this work. These results encourage further research into the performance of the XL marker under different shock etiologies.

## Supplementary Information

**Additional file 1: Table S1** ARRIVE guidelines 2.0 checklist.

**Additional file 2: Fig. S1** Study flow. The study flow describes the steps and procedure for each group. **Fig. S2** Gastric Impedance Spectroscopy (GIS) device. A—bedside monitor; B—sample applications in humans; C—feeding tube fitted with an impedance sensor.

**Additional file 3: Table S2** Blood gases, vital signs, and hemodynamic comparisons between the control group (CG) and the shock group (SG). Data presented as Median [IQR]; *p* for Kruskal–Wallis rank sum test. *BE* base excess, *Ca*^*2*+^ Ionized calcium, *CaO*_*2*_ Venous Oxygen Content, *Cl*^*−*^ Chlorine, *CO*_*2*_ Carbon Dioxide, *CvO*_*2*_ Arterial Oxygen Content, *DO*_*2*_ Oxygen Delivery, *FiO*_*2*_ Fraction of inspired Oxygen, *Hb* Hemoglobin, *K*^+^ Potassium, *MPAP* Mean Pulmonary Arterial Pressure, *Na*^+^ Sodium, *PvO*_*2*_ Partial Pressure of Oxygen in mixed venous blood, *PVR* Pulmonary Vascular Resistance, *REO*_*2*_ Oxygen Extraction Ratio, *RR* Respiratory Rate, *SaO*_*2*_ Arterial Oxygen Saturation, *SV* Stroke Volume, *SVR* Systemic Vascular Resistance, *VO*_*2*_ Oxygen Consumption. Events by shock criterion (MAP ≤ 48 mmHg) are T-2: 2 h before shock; T-1: 1 h before shock; T0: shock; T1: 1 h after shock; T2: 2 h after shock. **Figs. S3–S24** Comparison of relevant variables from Tables 1 and S2 between the control group (CG) and the shock group (SG).

**Additional file 4: Table S3** Summary of hematology, blood chemistry test, electrolyte panel, renal and liver panel. Data presented as Median [IQR]; *p* for Kruskal–Wallis rank sum test. * For statistically significant *p* values < 0.05. *ALP* Alkaline phosphatase, *ALT* Aminotransferase alanine, *AST* Aminotransferase aspartate, *CK* Creatine kinase, *K*^+^ Potassium, *LDH* Lactate dehydrogenase, *MCHC* Mean corpuscular hemoglobin concentration, *MCV* Mean corpuscular volume, *Na*^+^ Sodium, *PT* Prothrombin time, *PTT* Partial thromboplastin time.

**Additional file 5: Table S4** XL *p* values for post hoc Dunn´s all pairs test for Kruskal–Wallis rank sum test. Data presented as Median [IQR]; *CG* Control Group, *XL* gastric reactance. Events by shock criterion (MAP ≤ 48 mmHg) are T-2: 2 h before shock; T-1: 1 h before shock; T0: shock; T1: 1 h after shock; T2: 2 h after shock. * For statistically significant *p* values < 0.05. **Table S5** Lactate *p* values for post hoc Dunn´s all pairs test for Kruskal–Wallis rank sum test. Data presented as Median [IQR]; *CG* Control Group. Events by shock criterion (MAP ≤ 48 mmHg) are T-2: 2 h before shock; T-1: 1 h before shock; T0: shock; T1: 1 h after shock; T2: 2 h after shock. * For statistically significant *p* values < 0.05. **Table S6** XL_Min *p* values for post hoc Durbin–Conover test for Friedman Test. Data presented as Median [IQR]; CG Control Group, *XL_Min* minimum XL value per subject per event. Events by shock criterion (MAP ≤ 48 mmHg) are T-2: 2 h before shock; T-1: 1 h before shock; T0: shock; T1: 1 h after shock; T2: 2 h after shock. * For statistically significant *p* values < 0.05. **Table S7** Lac_Max *p* values for post hoc Durbin–Conover test for Friedman Test. Data presented as Median [IQR]; *CG* Control Group, *Lac_Max* maximum lactate value per subject per event. Events by shock criterion (MAP ≤ 48 mmHg) are T-2: 2 h before shock; T-1: 1 h before shock; T0: shock; T1: 1 h after shock; T2: 2 h after shock. * For statistically significant *p* values < 0.05.

**Additional file 6: Table S8** Endoscopic and histological examinations of the fundus, the body, and the antrum of the stomach. Data presented as Median [IQR]; *p* for Kruskal–Wallis rank sum test. * For statistically significant results (*p *< 0.05). The macroscopic appearance of the mucosa was classified by a gastroenterologist following the empirical: scale 0—normal mucosa, 1—stippling or epithelial hemorrhage, 2—pale mucosa, 3—violet mucosa, and 4—marmoreal mucosa. Biopsies were qualitatively classified by a pathologist as "Inflammation & Edema" and "Superficial Detachment" following the empirical scale: 0%, 12.5%, 25%, 50%, 75%, and 100%. Events by shock criterion (MAP ≤ 48 mmHg) are T-2: 2 h before shock; T-1: 1 h before shock; T0: shock; T1: 1 h after shock; T2: 2 h after shock. **Tables S9–S11** Control Group—endoscopic and histological examinations of the antrum, the body, and the fundus of subject C05**. Tables S12–S14** Shock Group—endoscopic and histological examinations of the antrum, the body, and the fundus of subject C11.

## Data Availability

The data sets used and analyzed during the current study are available from the corresponding author upon reasonable request.

## Abbreviations

ALP: Alkaline phosphatase
ALT: Alanine aminotransferase
ARRIVE: Animal Research Reporting of In Vivo Experiments
AST: Aspartate aminotransferase
AUC: Area under the curve
Ca^2+^: Ionized calcium
CBC: Complete blood count
CG: Control group
CI: Confidence interval
Cl^−^: Chloride
CO: Cardiac output
CO_2_: Carbon dioxide
CaO_2_: Arterial oxygen content
CvO_2_: Venous oxygen content
CVP: Central venous pressure
DO_2_: Oxygen delivery
BE: Base excess
FiO_2_: Fraction of inspired oxygen
GIS: Gastric impedance spectroscopy
GLP: Good Laboratory Practices
Hb: Hemoglobin
HR: Heart rate
K^+^: Potassium
Lac_Max: Maximum lactate value per subject per event
LDH: Lactate dehydrogenase
MAP: Mean arterial pressure
MODS: Multiple organ dysfunction syndrome
MPAP: Mean pulmonary artery pressure
Na^+^: Sodium
NPV: Negative predictive value
PAC: Pulmonary artery catheter
PAP: Pulmonary artery pressure
PADP: Pulmonary artery diastolic pressure
PASP: Pulmonary artery systolic pressure
PAWP: Pulmonary artery wedge pressure
PaCO_2_: Partial pressure of carbon dioxide in arterial blood
PEEP: Positive end-expiratory pressure
PO_2_: Partial pressure of oxygen
PvO_2_: Partial pressure of oxygen in mixed venous blood
PPV: Positive predictive value
PT: Prothrombin time
PTT: Activated partial thromboplastin time
PVR: Pulmonary vascular resistance
REO_2_: Oxygen extraction ratio
ROC: Receiver operating characteristic
RR: Respiratory rate
SD: Standard deviation
SG: Shock group
SaO_2_: Arterial oxygen saturation
SV: Stroke volume
SvO_2_: Mixed venous saturation
SVR: Systemic vascular resistance
TBV: Total blood volume
*V*O_2_: Oxygen consumption
VT: Tidal volume
*W*: Weight
WBC: White blood cell
XL: Gastric central reactance
XL_Min: Minimum XL value per subject per event
