# Supplementary material for: Comparison of gastric reactance with commonly used perfusion markers in a swine hypovolemic shock model
Source: Intensive Care Med Exp. 2022 Nov 18;10:49. doi: 10.1186/s40635-022-00476-1 (PMC9674824; doi:10.1186/s40635-022-00476-1)
Supplement: Supplementary file 2 — Additional file 2: Fig. S1 Study flow. The study flow describes the steps and procedure for each group. Fig. S2 Gastric Impedance Spectroscopy (GIS) device. A—bedside monitor; B—sample applications in humans; C—feeding tube fitted with an impedance sensor. [file 40635_2022_476_MOESM2_ESM.docx]

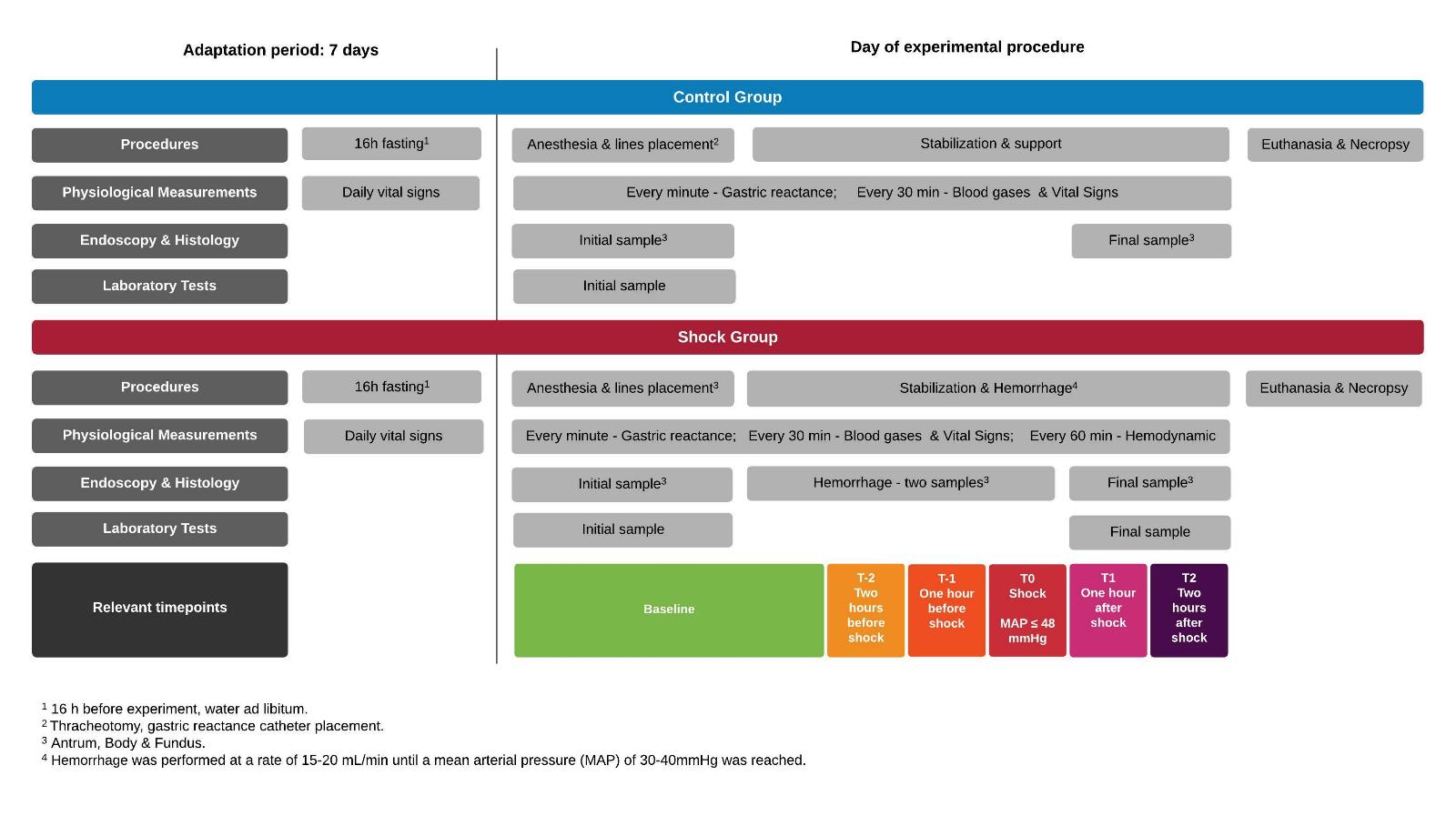


**Fig. S1** Study flow. The study flow displaying the steps and procedure for each group.

| 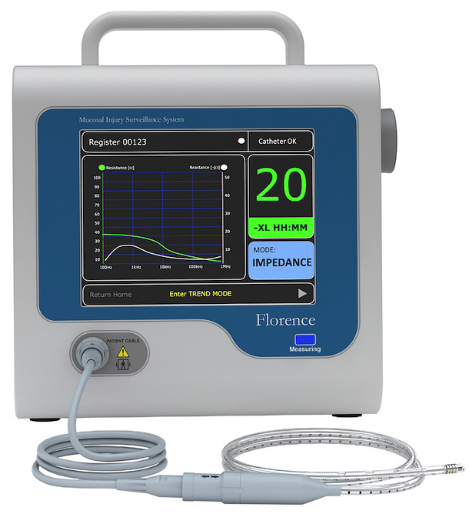  **A** | 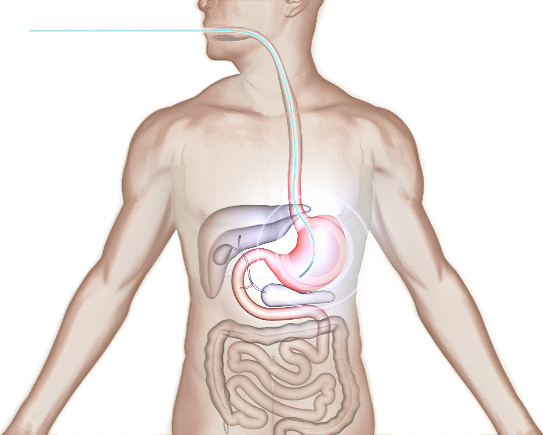  **B** |
| --- | --- |
| 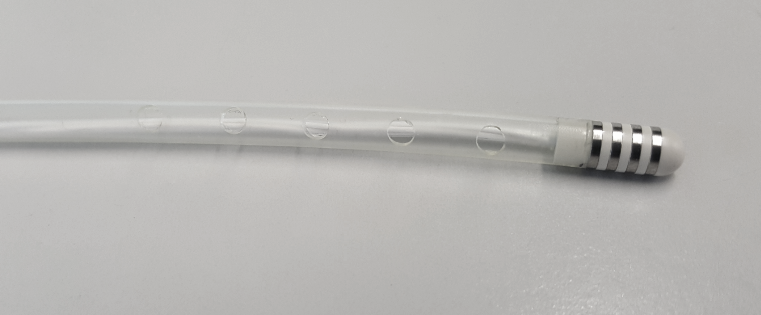  **C** | |

**Fig. S2** Gastric Impedance Spectroscopy (GIS) device. A – bedside monitor; B – sample applications in humans; C – feeding tube fitted with an impedance sensor.
