# Supplementary material for: Comparison of gastric reactance with commonly used perfusion markers in a swine hypovolemic shock model
Source: Intensive Care Med Exp. 2022 Nov 18;10:49. doi: 10.1186/s40635-022-00476-1 (PMC9674824; doi:10.1186/s40635-022-00476-1)
Supplement: Supplementary file 3 — Additional file 3: Table S2 Blood gases, vital signs, and hemodynamic comparisons between the control group (CG) and the shock group (SG). Data presented as Median [IQR]; p for Kruskal–Wallis rank sum test. BE base excess, Ca2+ Ionized calcium, CaO2 Venous Oxygen Content, Cl− Chlorine, CO2 Carbon Dioxide, CvO2 Arterial Oxygen Content, DO2 Oxygen Delivery, FiO2 Fraction of inspired Oxygen, Hb Hemoglobin, K+ Potassium, MPAP Mean Pulmonary Arterial Pressure, Na+ Sodium, PvO2 Partial Pressure of Oxygen in mixed venous blood, PVR Pulmonary Vascular Resistance, REO2 Oxygen Extraction Ratio, RR Respiratory Rate, SaO2 Arterial Oxygen Saturation, SV Stroke Volume, SVR Systemic Vascular Resistance, VO2 Oxygen Consumption. Events by shock criterion (MAP ≤ 48 mmHg) are T-2: 2 h before shock; T-1: 1 h before shock; T0: shock; T1: 1 h after shock; T2: 2 h after shock. Figs. S3–S24 Comparison of relevant variables from Tables 1 and S2 between the control group (CG) and the shock group (SG). [file 40635_2022_476_MOESM3_ESM.docx]

**Table S2.** Blood gases, vital signs, and hemodynamic comparisons between the control group (CG) and the shock group (SG).

|  | **Control Group** | | **Shock Group** | | | | | | |  |
| --- | --- | --- | --- | --- | --- | --- | --- | --- | --- | --- |
| **Variable** | **N = 62** | | **T-2**  **N = 31** | **T-1**  **N = 39** | **T0**  **N = 37** | | **T1**  **N = 36** | **T2**  **N = 21** | | ***p*** |
| ***Blood gases*** | | | | | | | | | | |
| BE (mmol/L) | 1.8 [1.0, 2.6] | | 2.2 [1.6, 3.5] | 2.1 [0.5, 3.3] | 0.7 [-1.0, 3.6] | | -3.5 [-8.7, 0.8] | -5.8 [-9.0, -3.7] | | <0.001* |
| K^+^ (mmol/L) | 4.5 [4.3, 4.9] | | 4.5 [4.1, 5.1] | 5.1 [4.6, 5.8] | 5.7 [5.4, 6.7] | | 6.8 [6.2, 8.3] | 6.7 [6.3, 7.7] | | <0.001* |
| Na^+^ (mmol/L) | 134.0 [132.0, 135.0] | | 135.0 [130.5, 138.0] | 134.0 [129.0, 137.0] | 133.0 [129.5, 135.0] | | 133.0 [127.5, 135.0] | 133.0 [128.0, 135.0] | | 0.105 |
| Ca^2+^ (mmol/L) | 1.2 [1.1, 1.3] | | 1.3 [1.1, 1.4] | 1.2 [1.0, 1.3] | 1.2 [1.1, 1.3] | | 1.1 [1.0, 1.3] | 1.1 [1.1, 1.2] | | 0.045* |
| Cl^-^ (mmol/L) | 100.0 [99.0, 101.0] | | 101.0 [98.0, 105.0] | 100.0 [97.5, 103.5] | 100.0 [98.0, 103.0] | | 101.0 [97.8, 103.0] | 102.0 [100.0, 104.0] | | 0.401 |
| Glucose (mg/dL) | 102.0 [91.0, 124.5] | | 97.0 [67.5, 109.5] | 105.0 [91.5, 119.5] | 95.0 [85.2, 113.8] | | 88.0 [59.5, 110.5] | 82.0 [78.0, 110.0] | | 0.070 |
| Hb (g/dL) | 8.8 [8.6, 9.2] | | 8.8 [7.9, 9.9] | 9.2 [8.4, 9.7] | 9.0 [8.4, 9.6] | | 9.1 [8.1, 9.6] | 9.6 [9.0, 9.8] | | 0.294 |
| ***Vital Signs*** | | | | | | | | | | |
| RR (rpm) | 20.0 (20.0, 24.0] | | 22.0 (20.0, 25.0] | 22.0 (20.0, 24.0] | 22.0 (20.0, 24.0] | | 22.0 (20.0, 28.0] | 22.0 (20.0, 26.0] | | 0.006* |
| ***Hemodynamic & Oxygenation*** | | | | | | | | | | |
| MPAP (mmHg) | | - | 20.0 [17.0, 23.8] | 20.5 [17.0, 24.2] | 19.0 [14.5, 21.5] | 21.0 [16.0, 25.8] | | 22.0 [15.0, 26.0] | 0.255 | |
| SVR (din-s/cm^5^) | | - | 967.0 [941.2, 1,192.8] | 1,131.0 [773.5, 1,182.8] | 895.0 [758.0, 967.5] | 773.0 [691.0, 913.0] | | 800.0 [667.0, 889.0] | 0.013* | |
| PVR (din-s/cm5) | | - | 179.5 [133.0, 237.0] | 290.0 [188.5, 352.8] | 285.0 [176.0, 391.5] | 345.5 [261.8, 489.0] | | 400.0 [282.0, 552.0] | <0.001* | |
| SV (mL/beat) | | - | 30.0 [26.5, 39.0] | 27.0 [20.0, 37.2] | 22.0 [16.0, 33.0] | 17.0 [15.0, 22.8] | | 19.0 [17.0, 24.0] | <0.001* | |
| SaO_2_ (%) | | - | 98.5 [94.0, 99.0] | 98.0 [97.8, 99.0] | 98.0 [96.0, 99.0] | 96.0 [93.5, 98.0] | | 96.0 [88.0, 97.0] | <0.001* | |
| DO_2_ (mL/min) | | - | 387.0 [339.0, 506.5] | 381.5 [303.0, 489.0] | 367.0 [273.0, 410.0] | 283.5 [270.0, 389.8] | | 333.0 [270.0, 383.0] | 0.013* | |
| VO_2_ (mL/min) | | - | 127.5 [115.0, 167.8] | 138.0 [105.2, 178.0] | 147.0 [117.5, 184.0] | 175.0 [103.0, 241.2] | | 154.0 [95.0, 194.0] | 0.466 | |
| PvO_2_ (mmHg) | | - | 40.0 [40.0, 48.2] | 40.0 [40.0, 46.0] | 40.0 [40.0, 46.0] | 40.0 [40.0, 40.0] | | 40.0 [40.0, 40.0] | 0.203 | |
| FiO_2_ (%) | | - | 0.8 [0.8, 75.0] | 35.4 [0.8, 75.0] | 0.8 [0.8, 75.0] | 0.8 [0.8, 0.8] | | 0.8 [0.8, 0.8] | 0.036* | |
| CO_2_ (mmHg) | | - | 157.0 [124.2, 181.0] | 139.5 [119.0, 183.0] | 150.0 [122.0, 186.0] | 146.5 [85.0, 215.2] | | 165.5 [128.2, 193.0] | 0.966 | |
| CvO_2_ (mL/dL) | | - | 8.0 [6.0, 8.8] | 8.0 [6.0, 9.0] | 7.0 [6.0, 8.0] | 6.5 [5.2, 7.8] | | 7.0 [6.0, 8.0] | 0.130 | |
| CaO_2_ (mL/dL) | | - | 12.0 [11.2, 13.0] | 12.0 [12.0, 13.0] | 12.0 [11.5, 13.0] | 12.0 [11.0, 13.0] | | 12.0 [11.0, 13.0] | 0.778 | |
| REO_2_ (%) | | - | 33.8 [25.3, 48.3] | 38.7 [26.0, 50.8] | 43.0 [39.1, 50.5] | 40.3 [36.7, 55.1] | | 40.1 [35.8, 50.7] | 0.059 | |
| Data presented as Median [IQR]; *p* for Kruskal-Wallis rank sum test. *for statistically significant results (*p* < 0.05)  *BE* base excess, *Ca^2+^* Ionized calcium, *CaO_2_* Venous Oxygen Content, *Cl^-^* Chlorine, *CO_2_* Carbon Dioxide, *CvO_2_* Arterial Oxygen Content, *DO_2_* Oxygen Delivery, *FiO_2_* Fraction of inspired Oxygen, *Hb* Hemoglobin, *K^+^* Potassium, *MPAP* Mean Pulmonary Arterial Pressure, *Na^+^* Sodium, *PvO_2_* Partial Pressure of Oxygen in mixed venous blood, *PVR* Pulmonary Vascular Resistance, *REO_2_* Oxygen Extraction Ratio, *RR* Respiratory Rate, *SaO_2_* Arterial Oxygen Saturation, *SV* Stroke Volume, *SVR* Systemic Vascular Resistance, *VO_2_* Oxygen Consumption. Events by shock criterion (MAP ≤ 48 mmHg) T-2: two hours before shock; T-1:one hour before shock; T0: shock; T1: one hour after hypovolemic shock; T2: two hours after shock. | | | | | | | | | | |

***Fig. S 1 Fig. S 2***

**Blood gases**

| **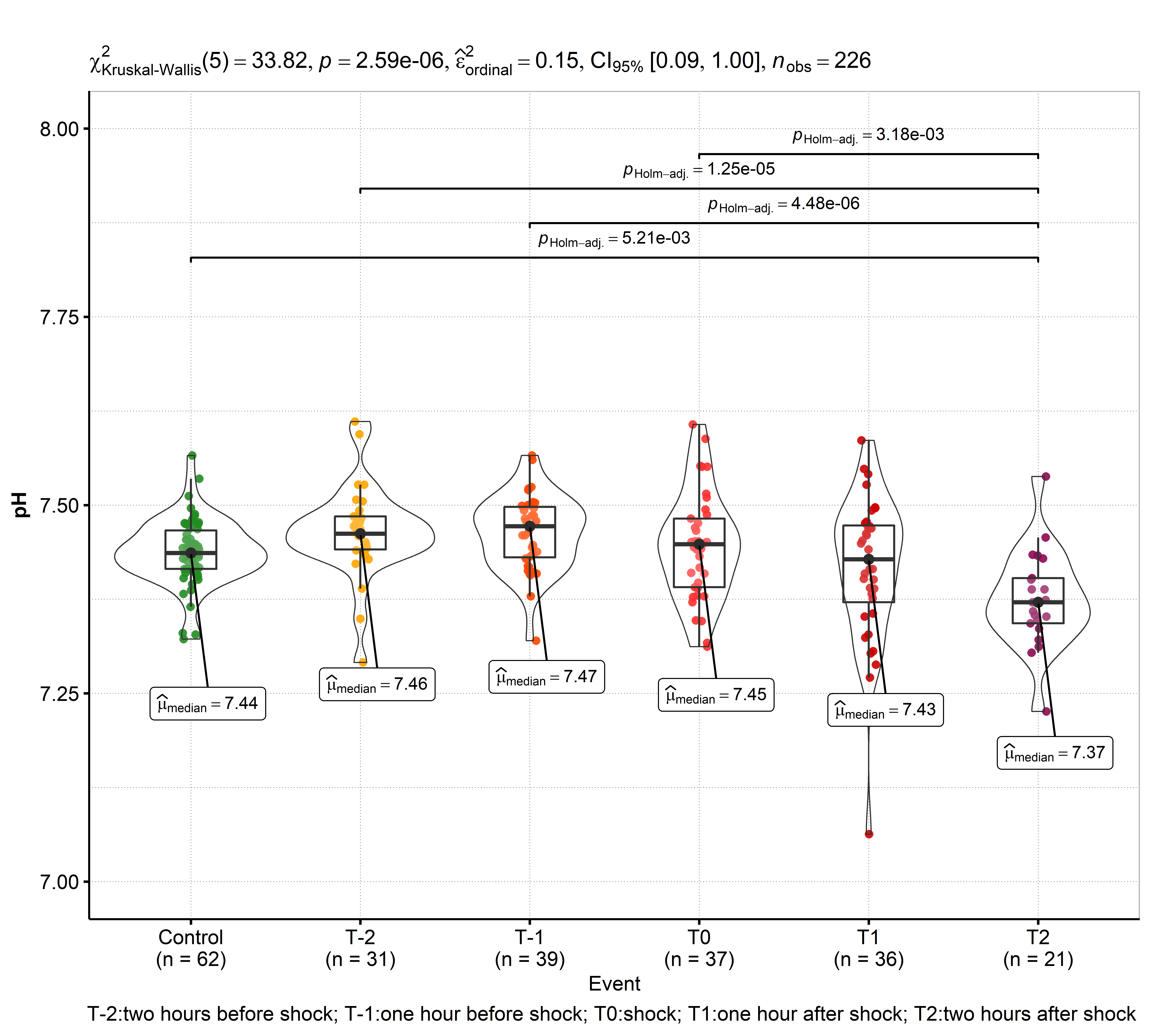**  **Fig. S3** Comparison of pH between the control group (CG) and the shock group (SG). |  | 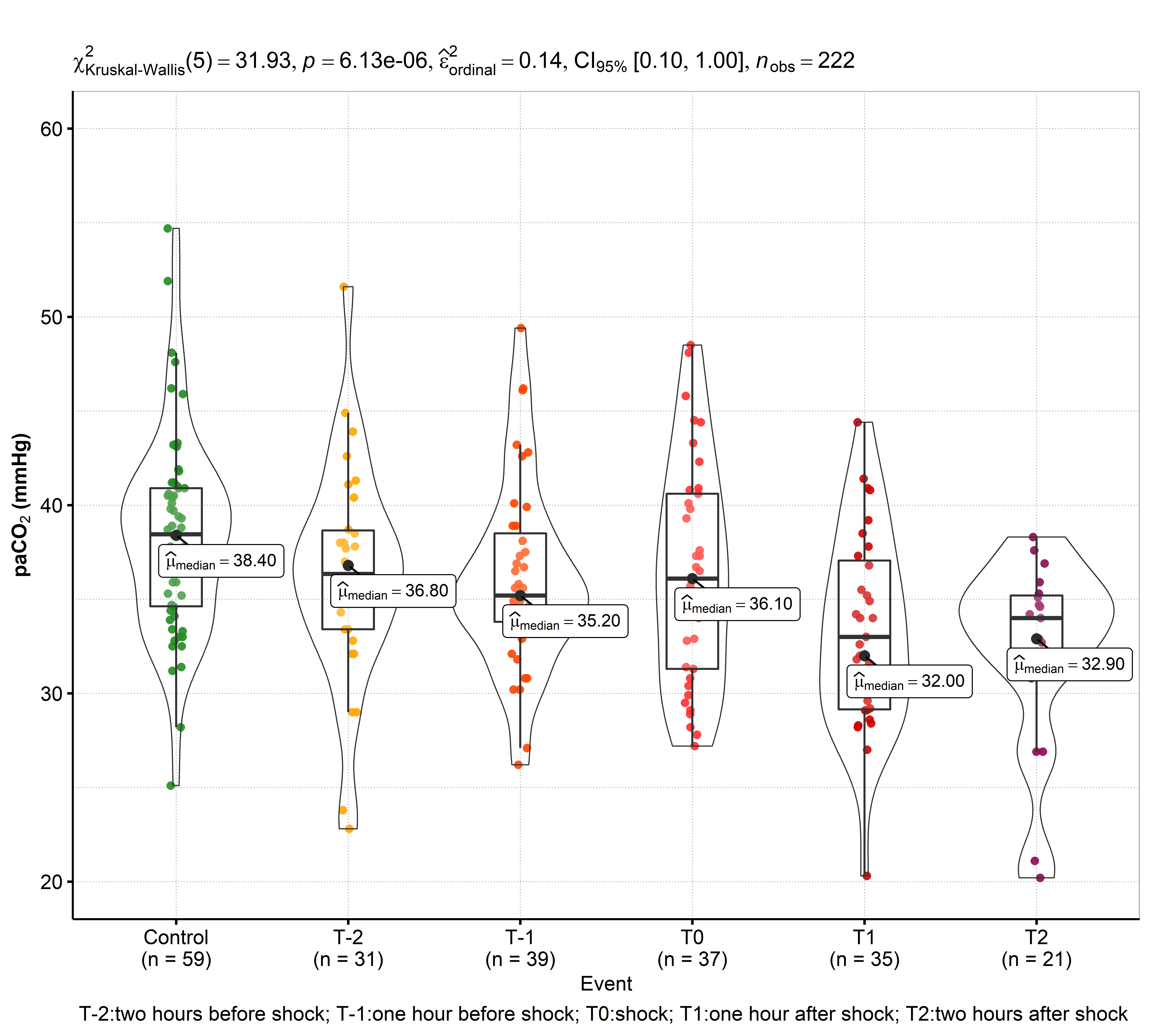  **Fig. S4** Comparison of partial pressure of carbon dioxide in arterial blood (paCO_2_) between the control group (CG) and the shock group (SG). |
| --- | --- | --- |

| 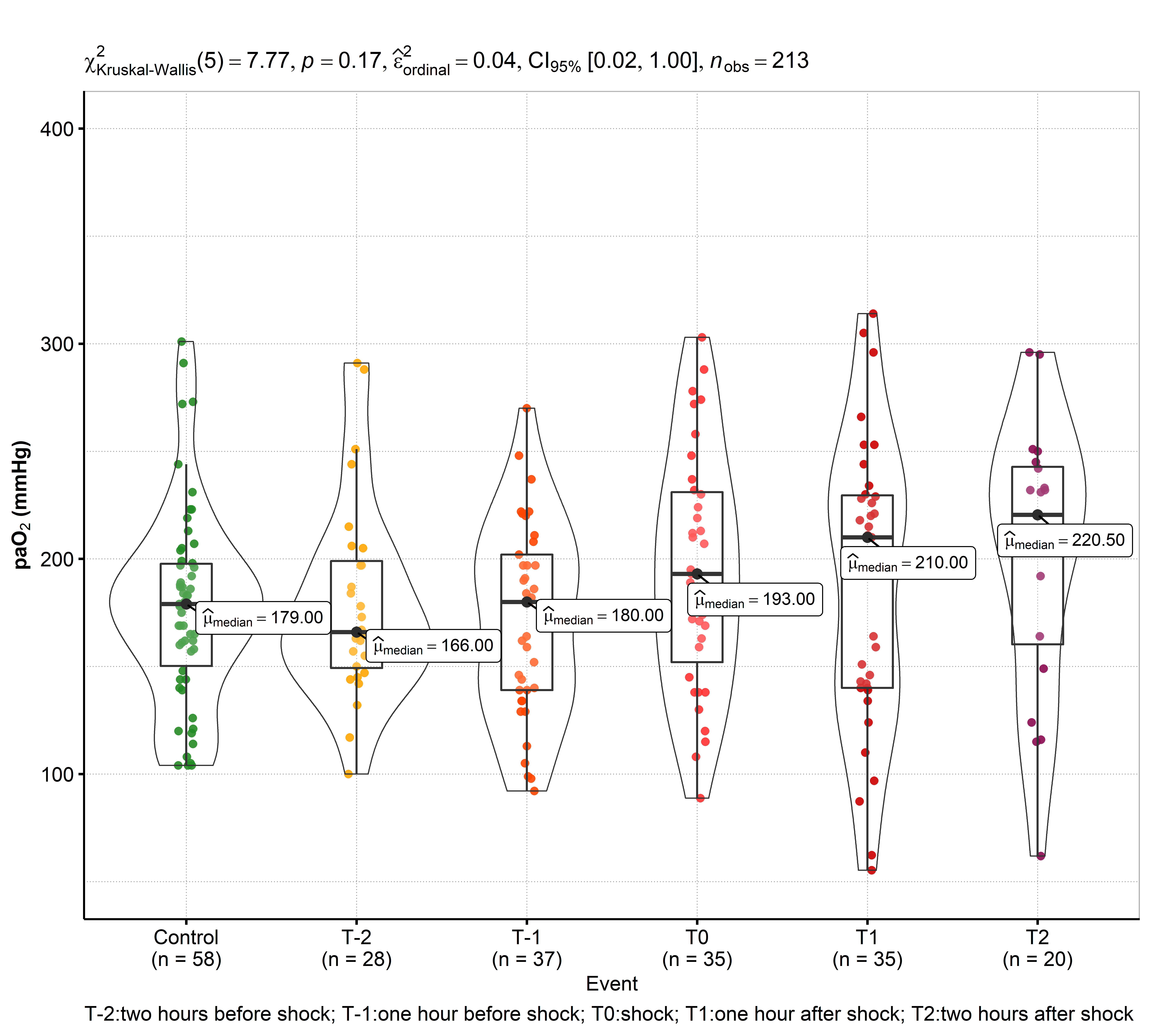  **Fig. S5** Comparison of partial pressure of oxygen (paO_2_) between the control group (CG) and the shock group (SG). |  | 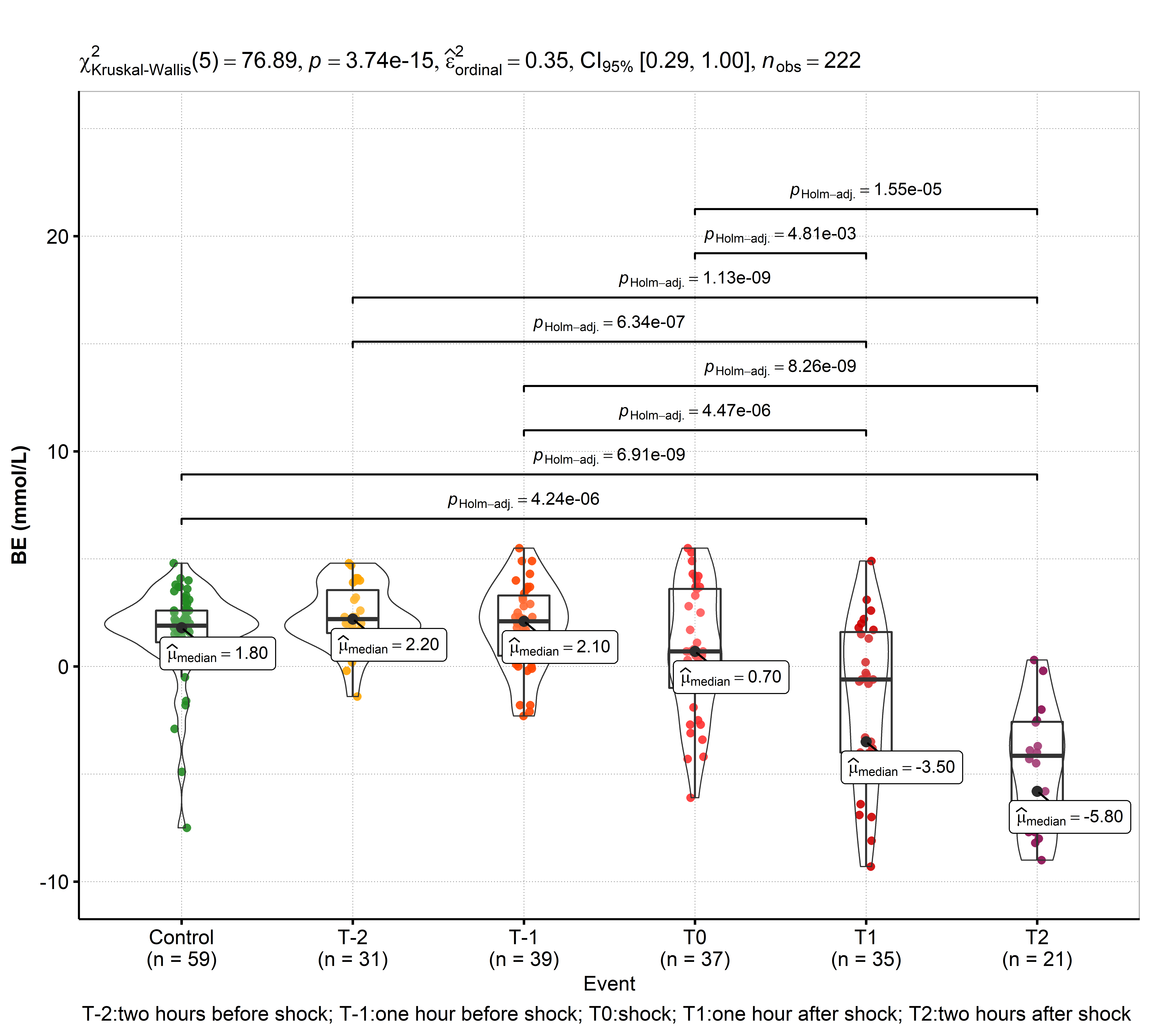  **Fig. S6** Comparison of base excess (BE) between the control group (CG) and the shock group (SG). |
| --- | --- | --- |

| **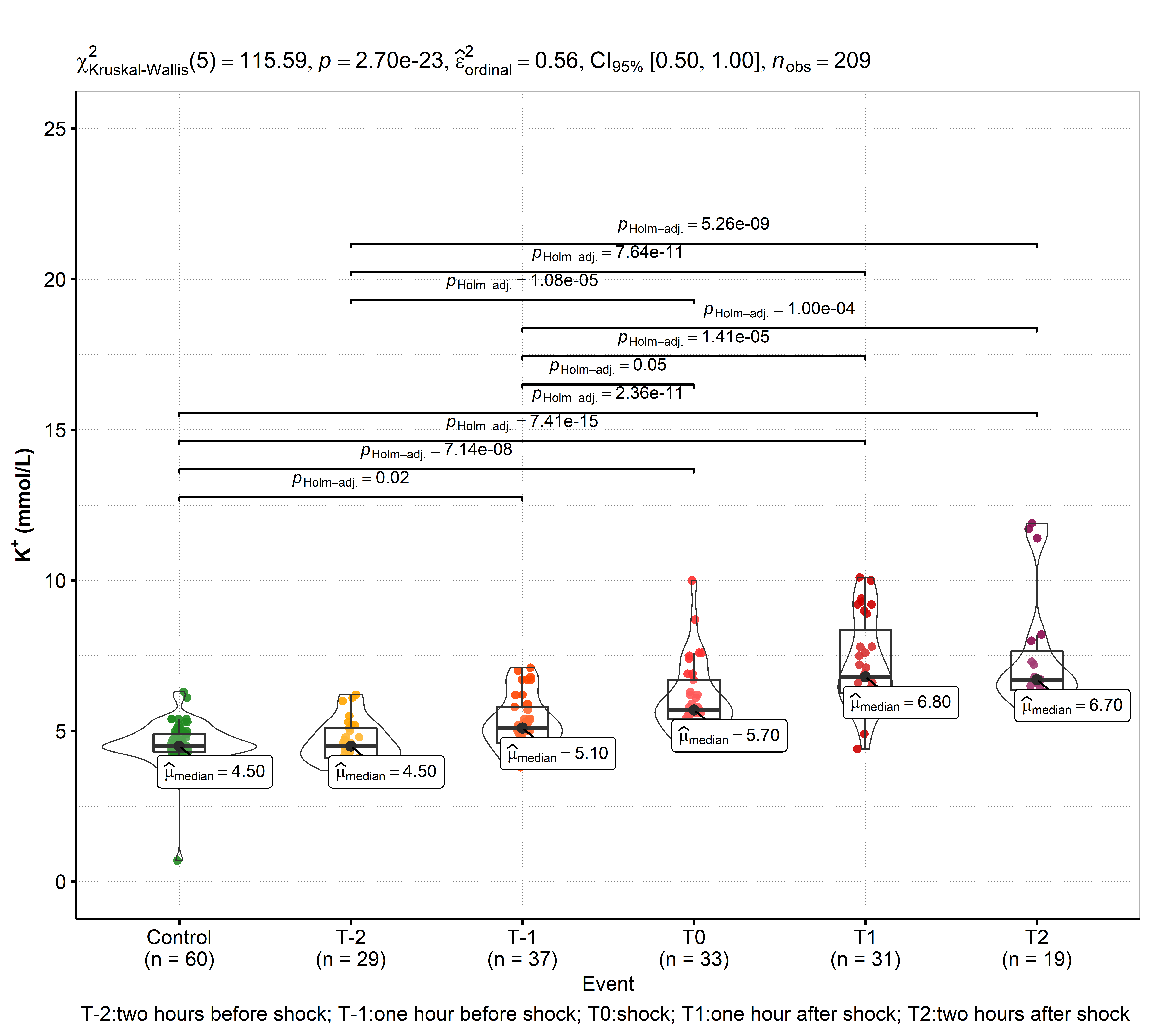**  **Fig. S7**  Comparison of potassium (K^+^) between the control group (CG) and the shock group (SG). |  | **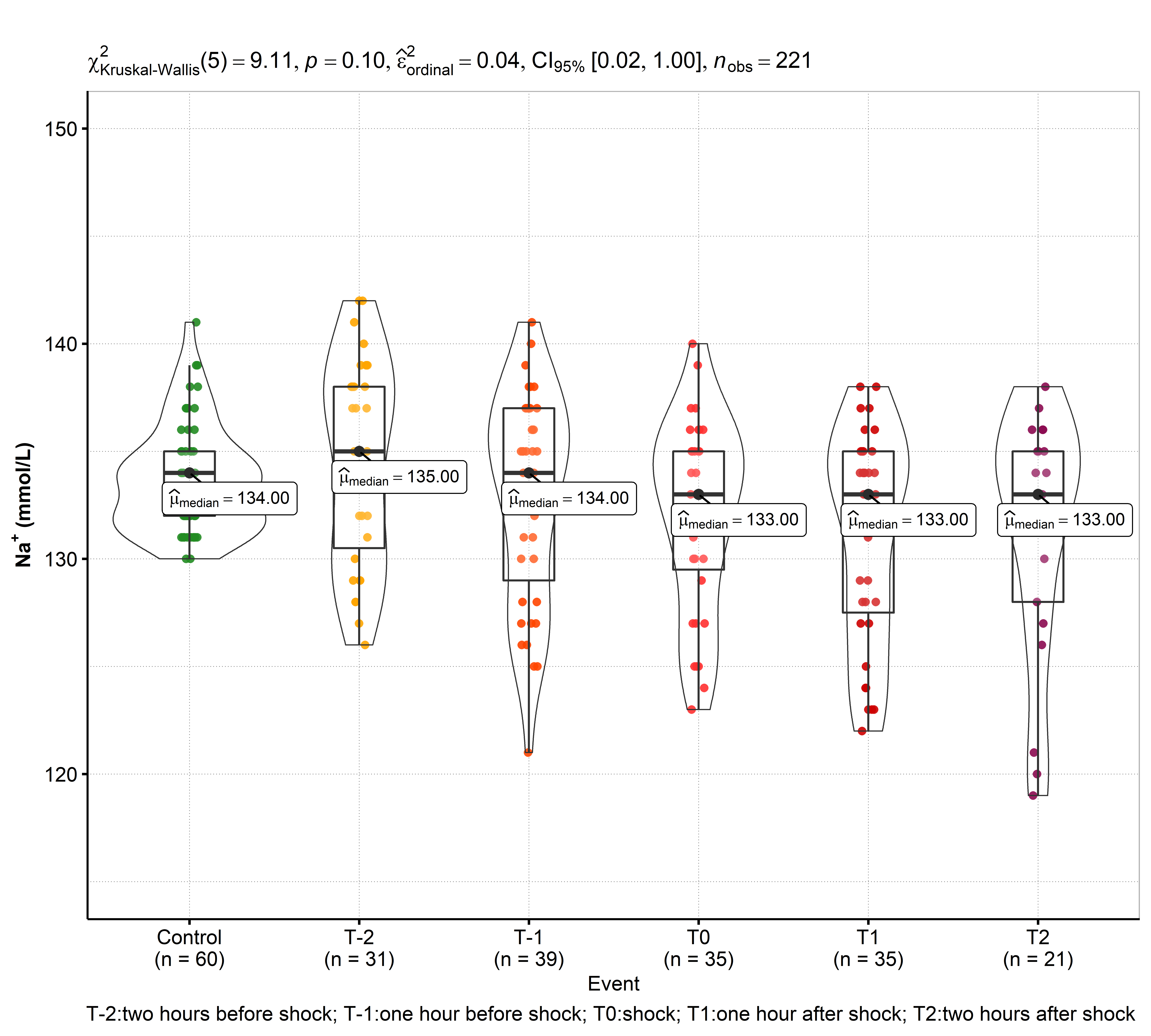**  **Fig. S8**  Comparison of sodium (Na^+^) between the control group (CG) and the shock group (SG). |
| --- | --- | --- |

| **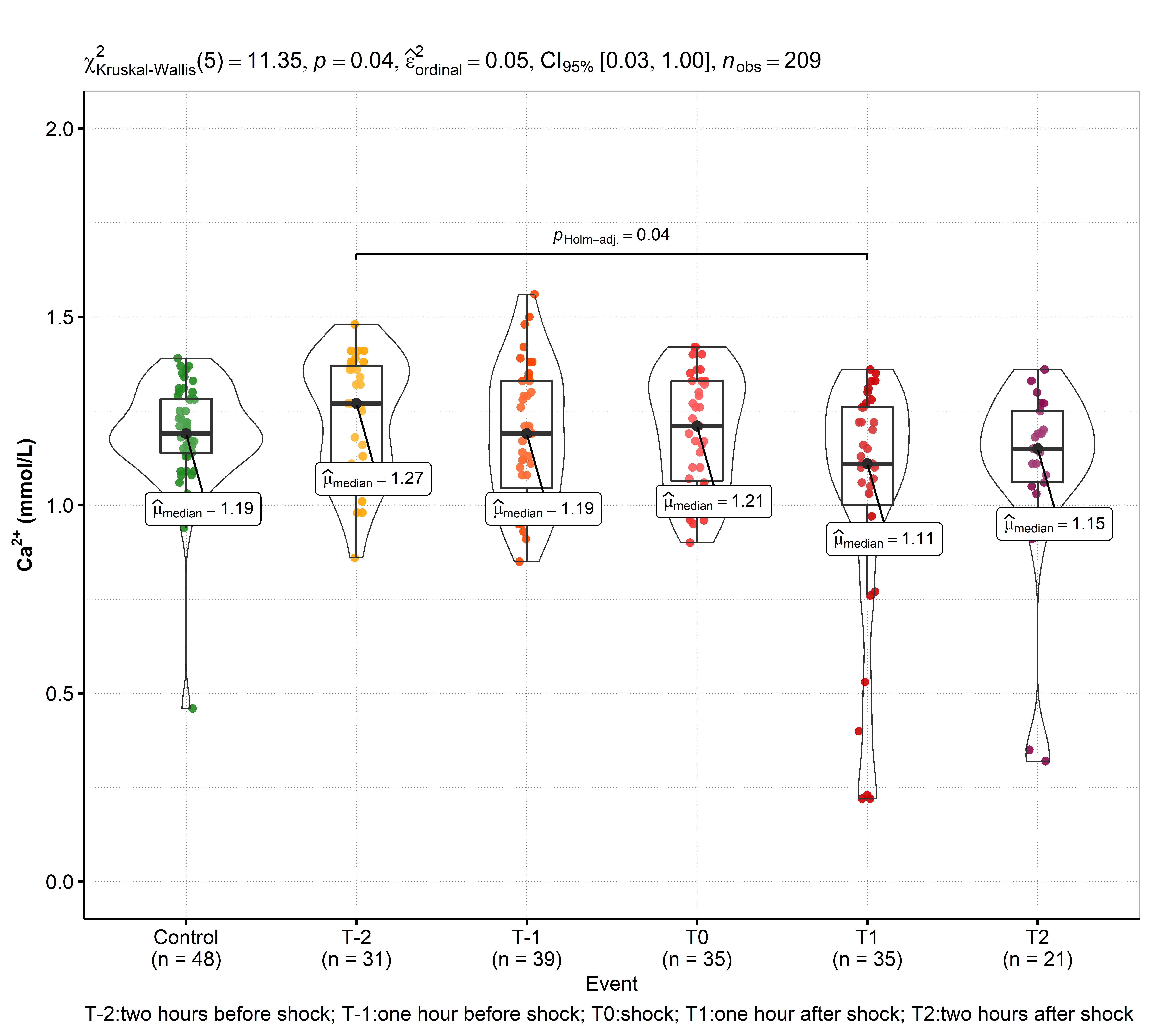**  **Fig. S9**  Comparison of ionized calcium (Ca^2+^) between the control group (CG) and the shock group (SG). |  | **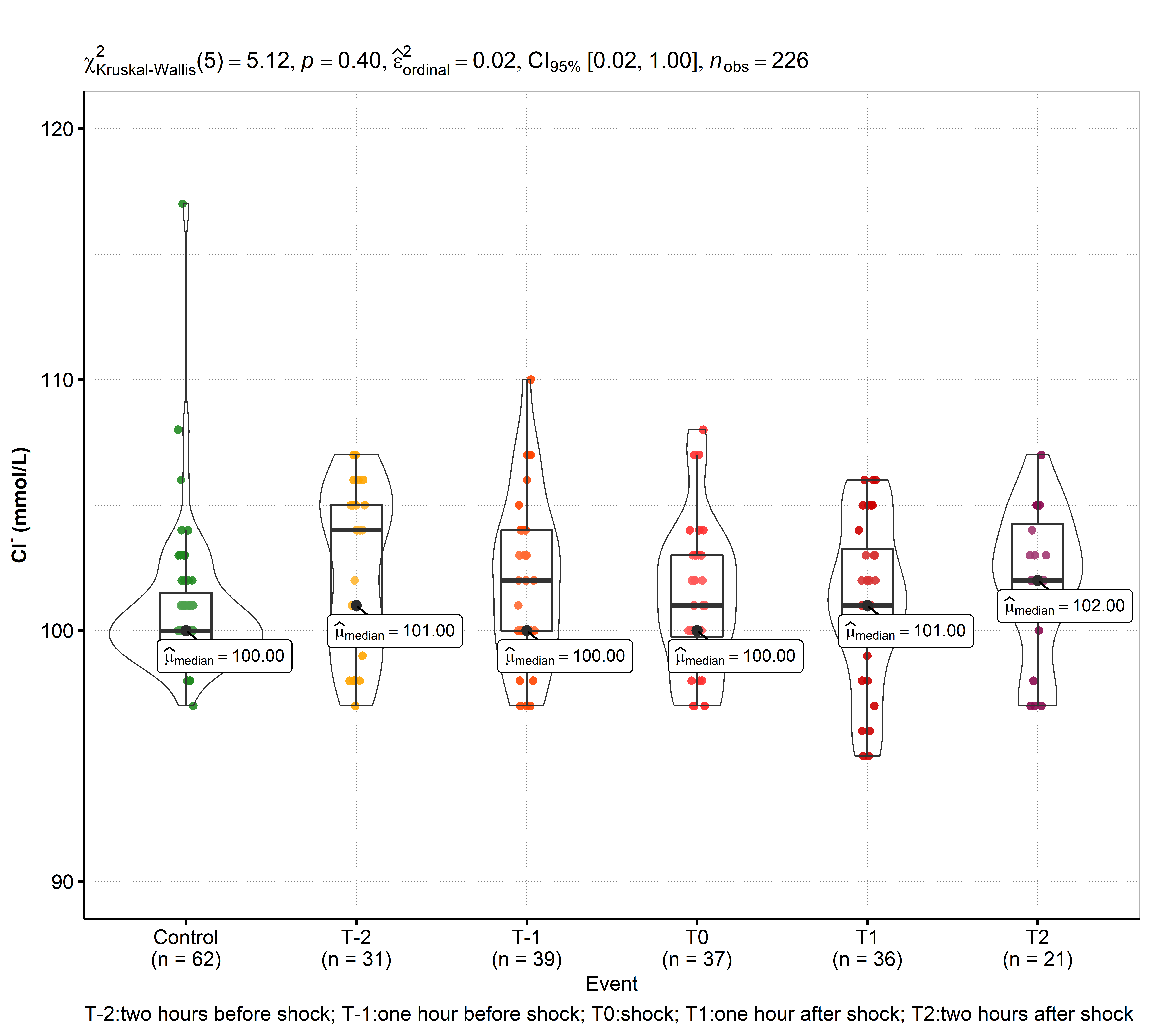**  **Fig. S10**  Comparison of chloride (Cl^-^)between the control group (CG) and the shock group (SG). |
| --- | --- | --- |

| 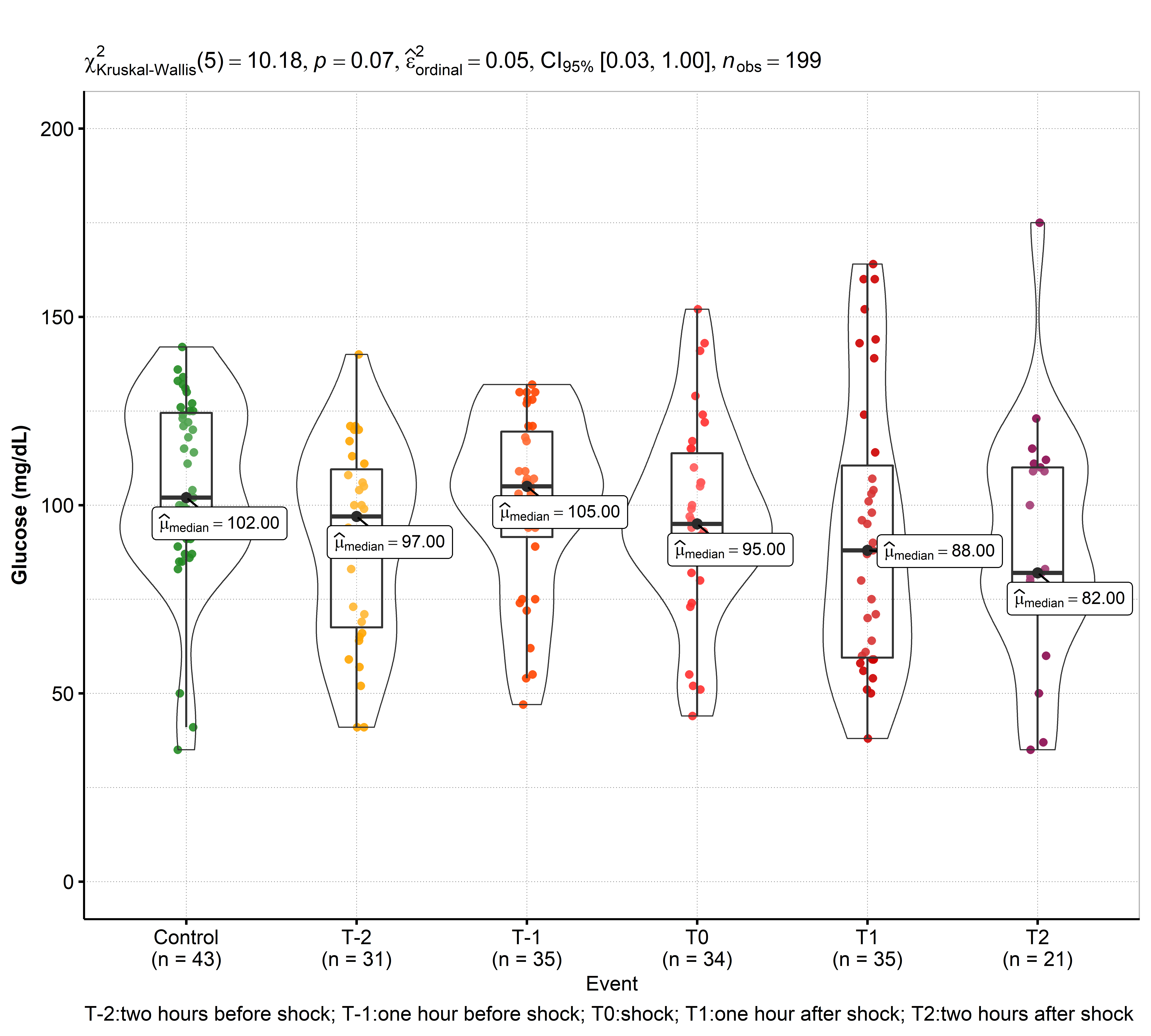  **Fig. S11** Comparison of glucose between the control group (CG) and the shock group (SG). |  | 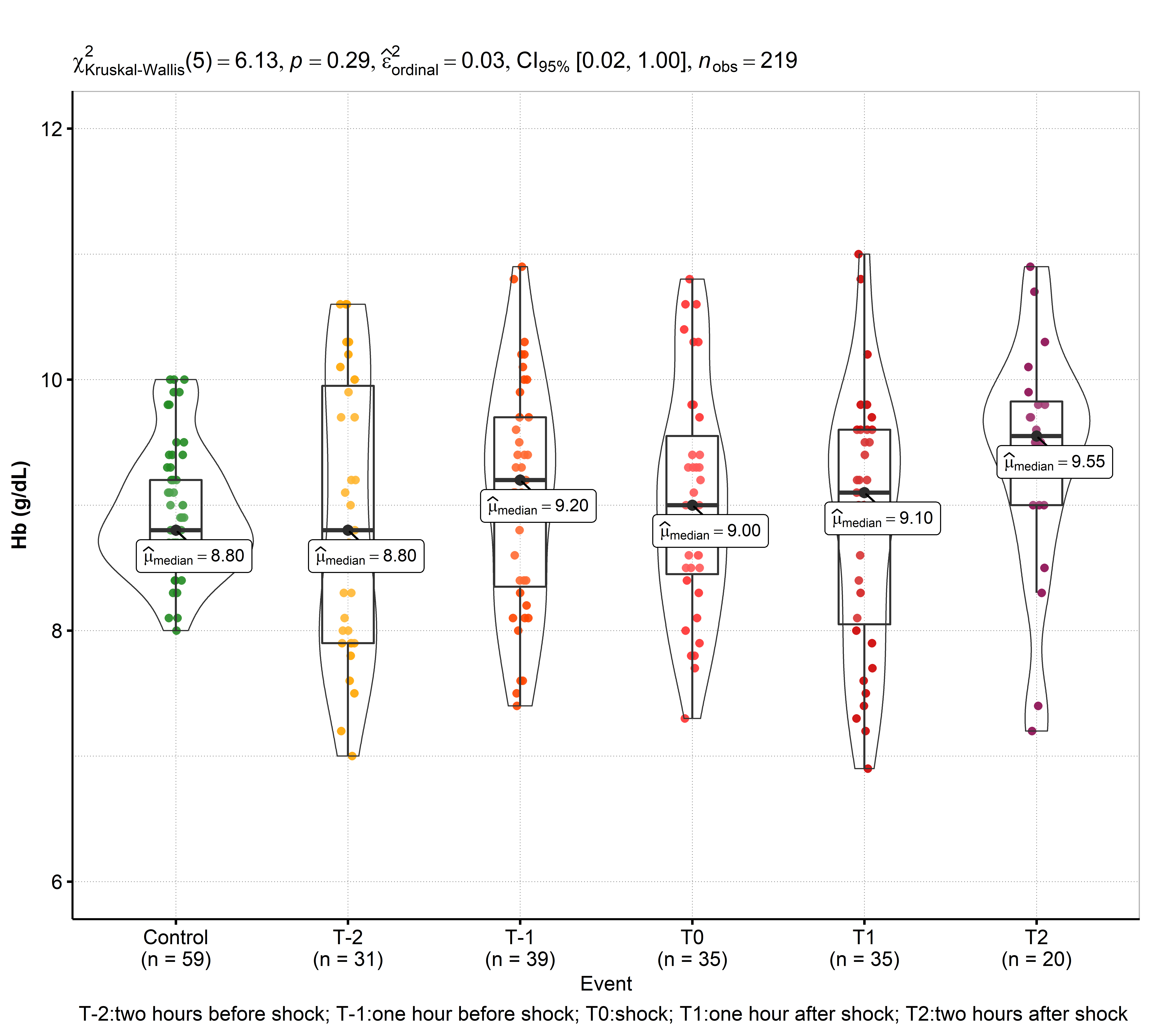  **Fig. S12** Comparison of hemoglobin (Hb) between the control group (CG) and the shock group (SG). |
| --- | --- | --- |

**Vital Signs**

| **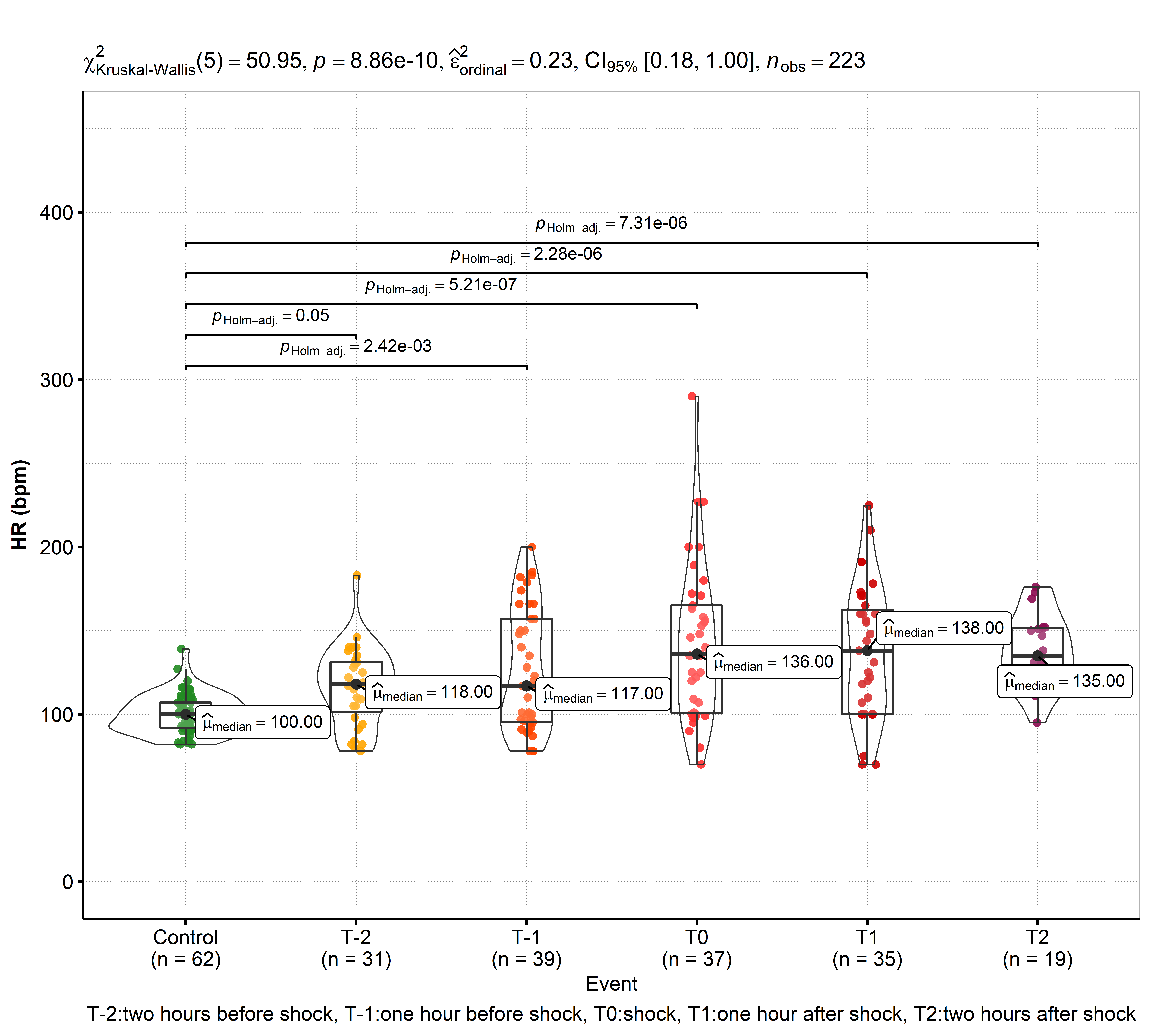**  **Fig. S13**  Comparison of heart rate (HR) between the control group (CG) and the shock group (SG). |  | **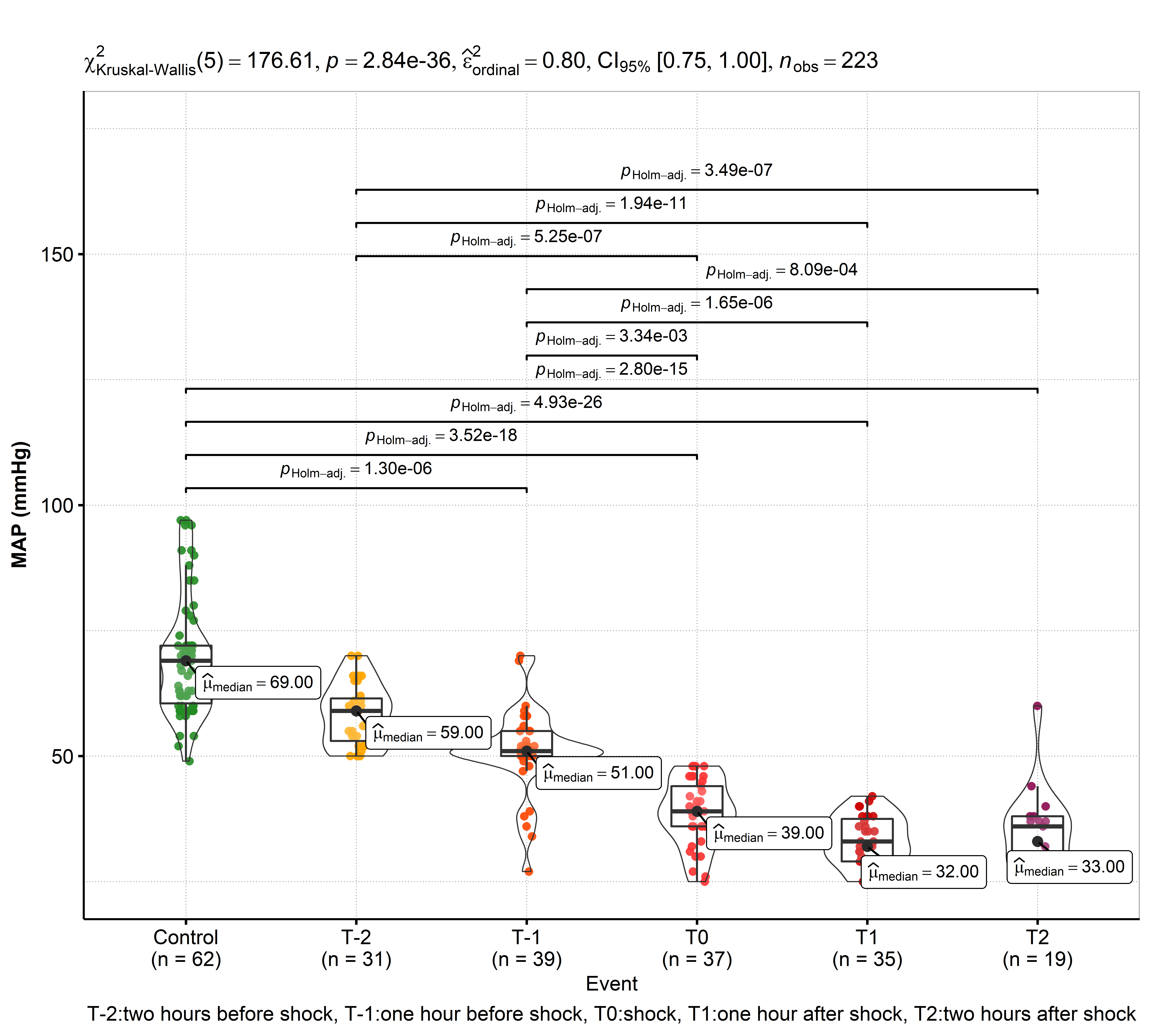**  **Fig. S14**  Comparison of mean arterial pressure (MAP) between the control group (CG) and the shock group (SG). |
| --- | --- | --- |

| **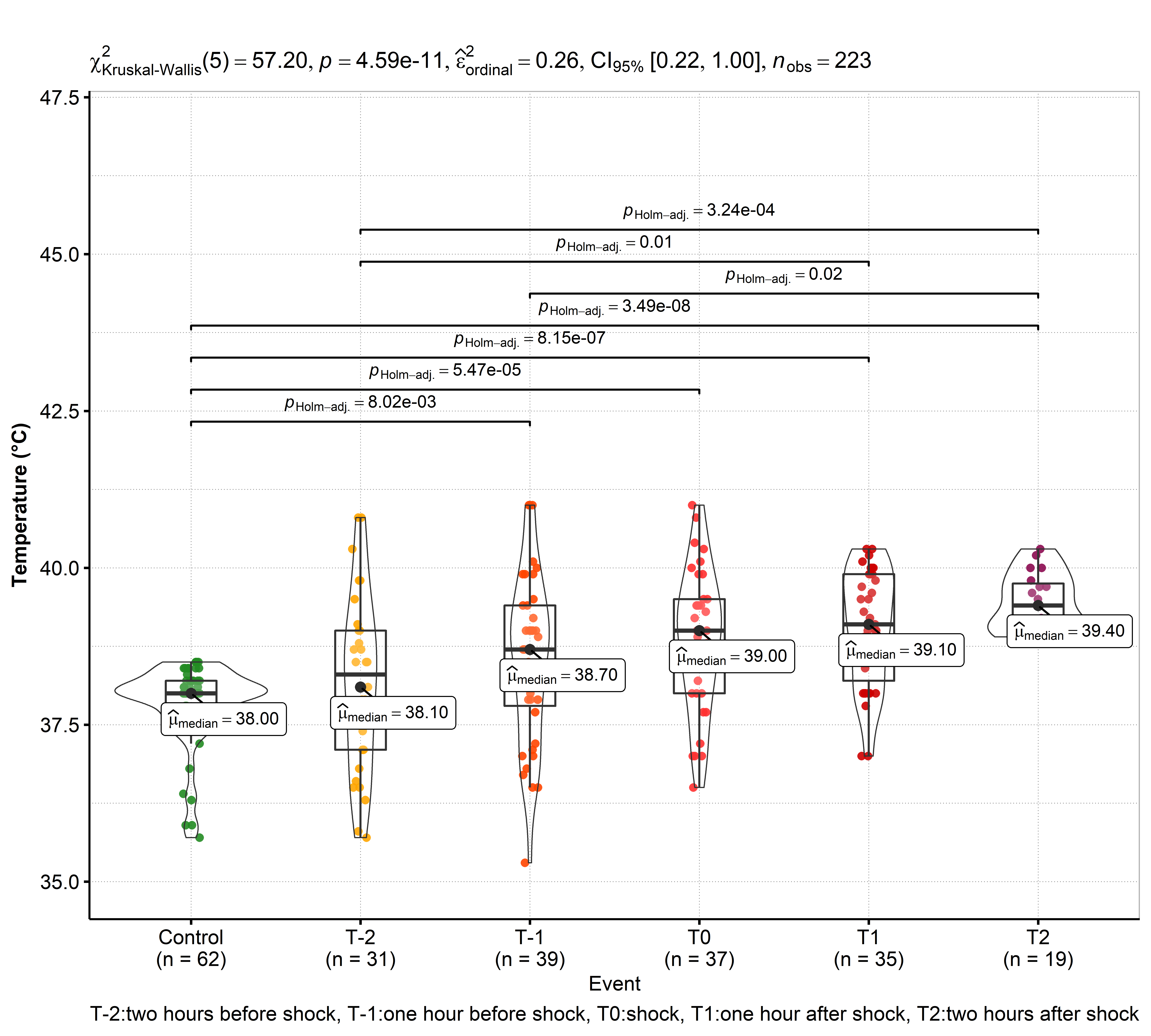**  **Fig. S15**  Comparison of temperature between the control group (CG) and the shock group (SG). |  | **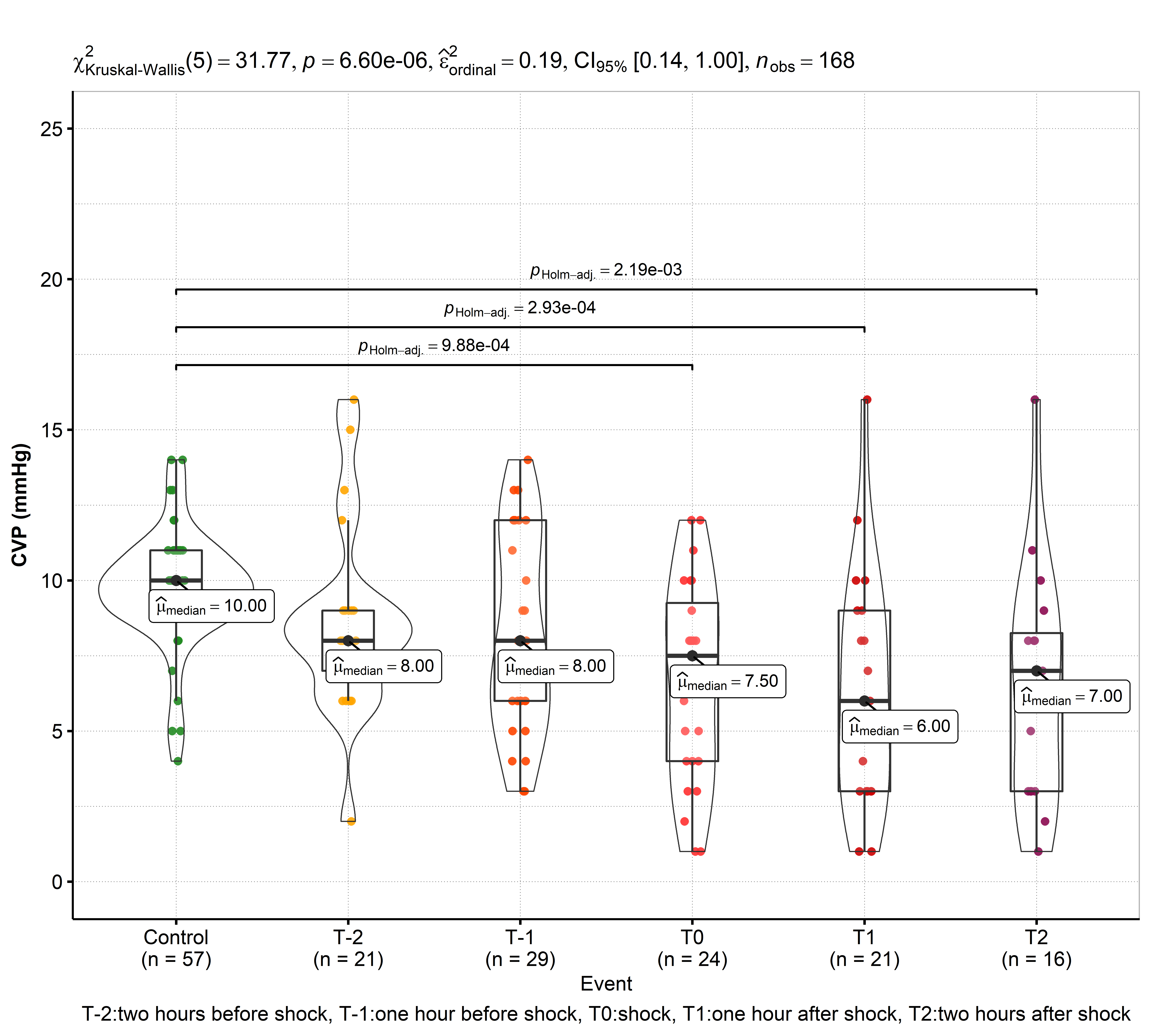**  **Fig. S16**  Comparison of central venous pressure (CVP) between the control group (CG) and the shock group (SG). |
| --- | --- | --- |

**
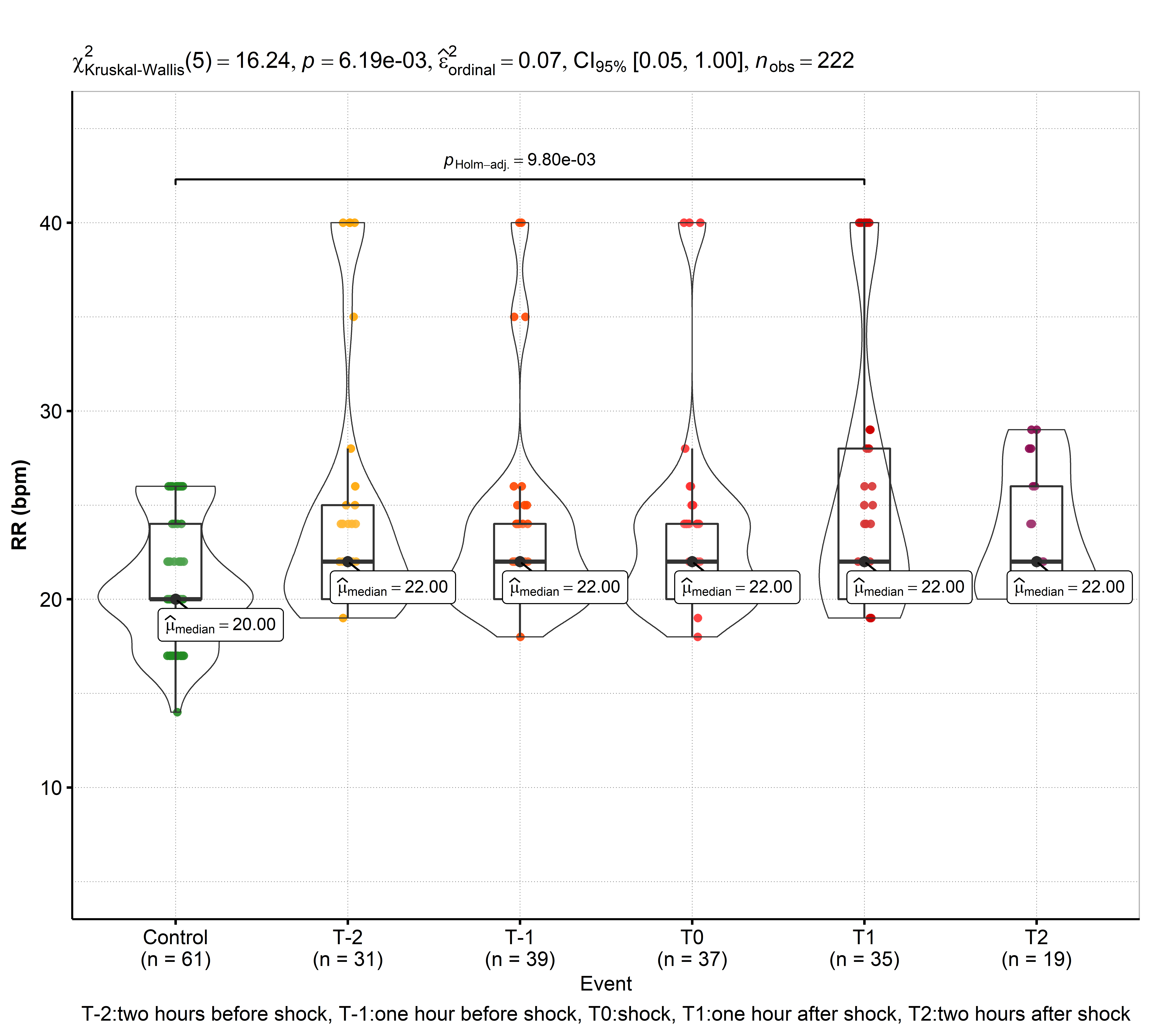
**

**Fig. S17**  Comparison of respiratory rate (RR) between the control group (CG) and the shock group (SG).

**Haemodynamic & Oxygenation**

| 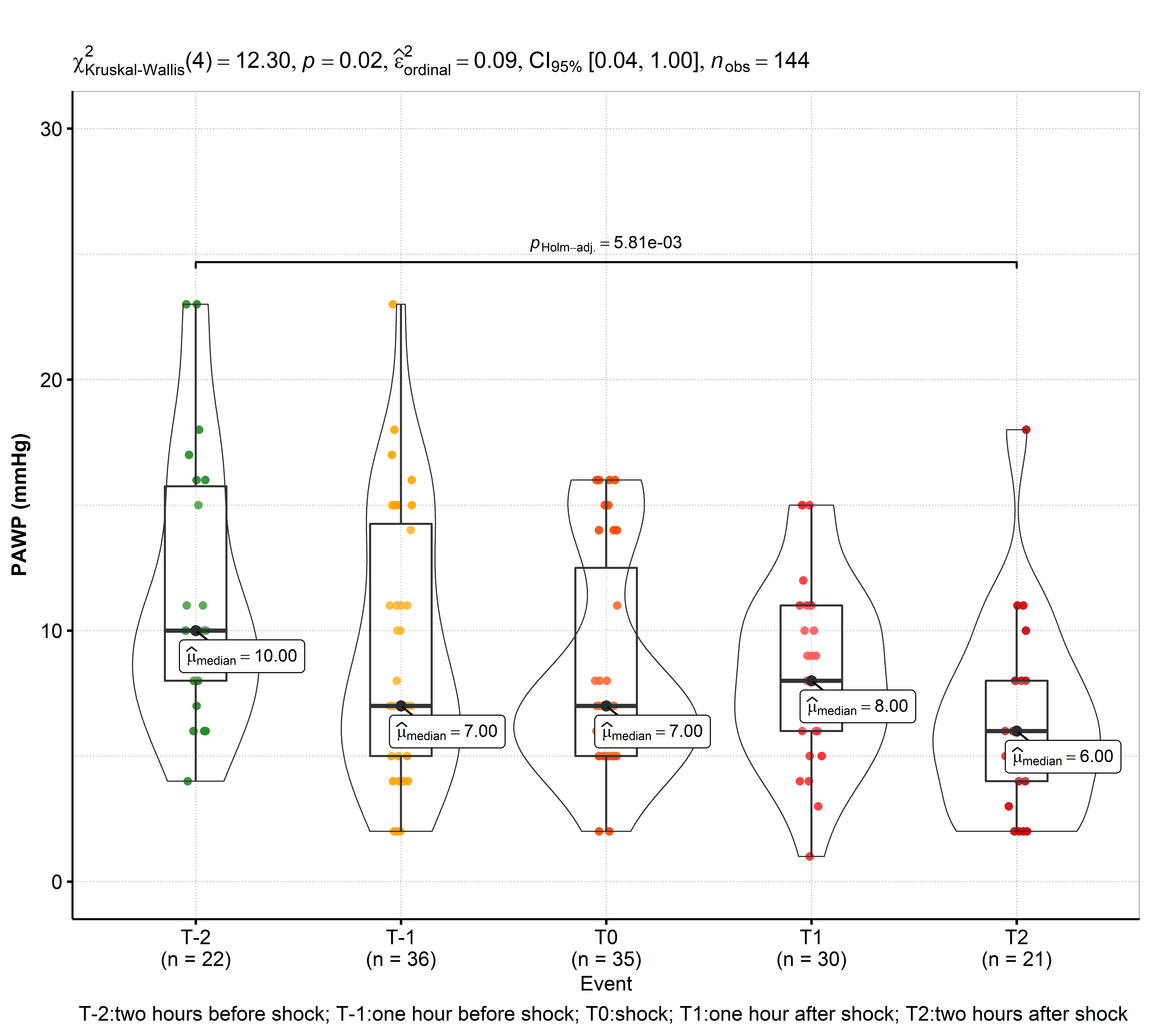  **Fig. S18**  Comparison of pulmonary artery wedge (PAWP) pressure between the control group (CG) and the shock group (SG). |  | 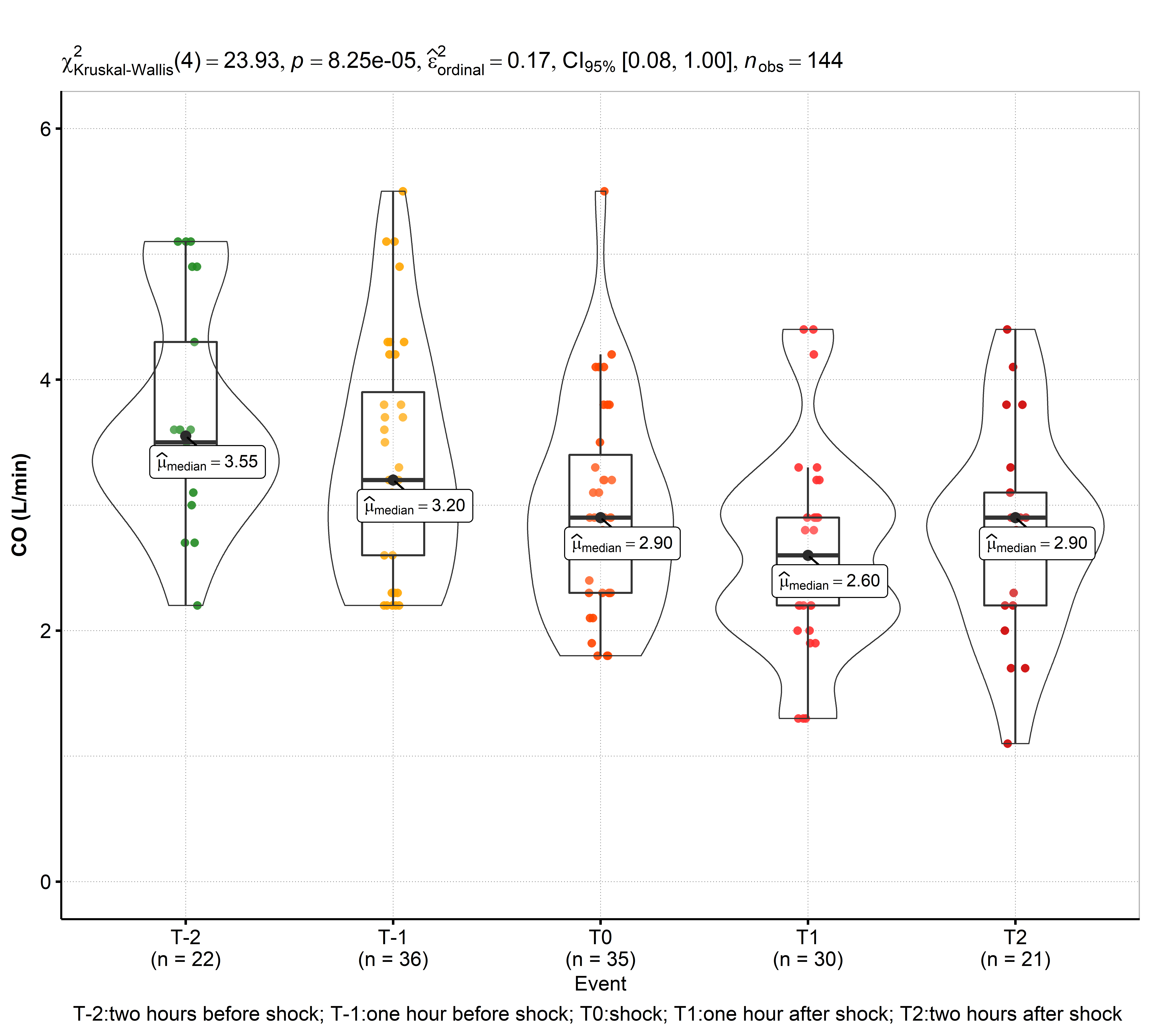  **Fig. S19**  Comparison of cardiac output (CO) between the control group (CG) and the shock group (SG). |
| --- | --- | --- |

| 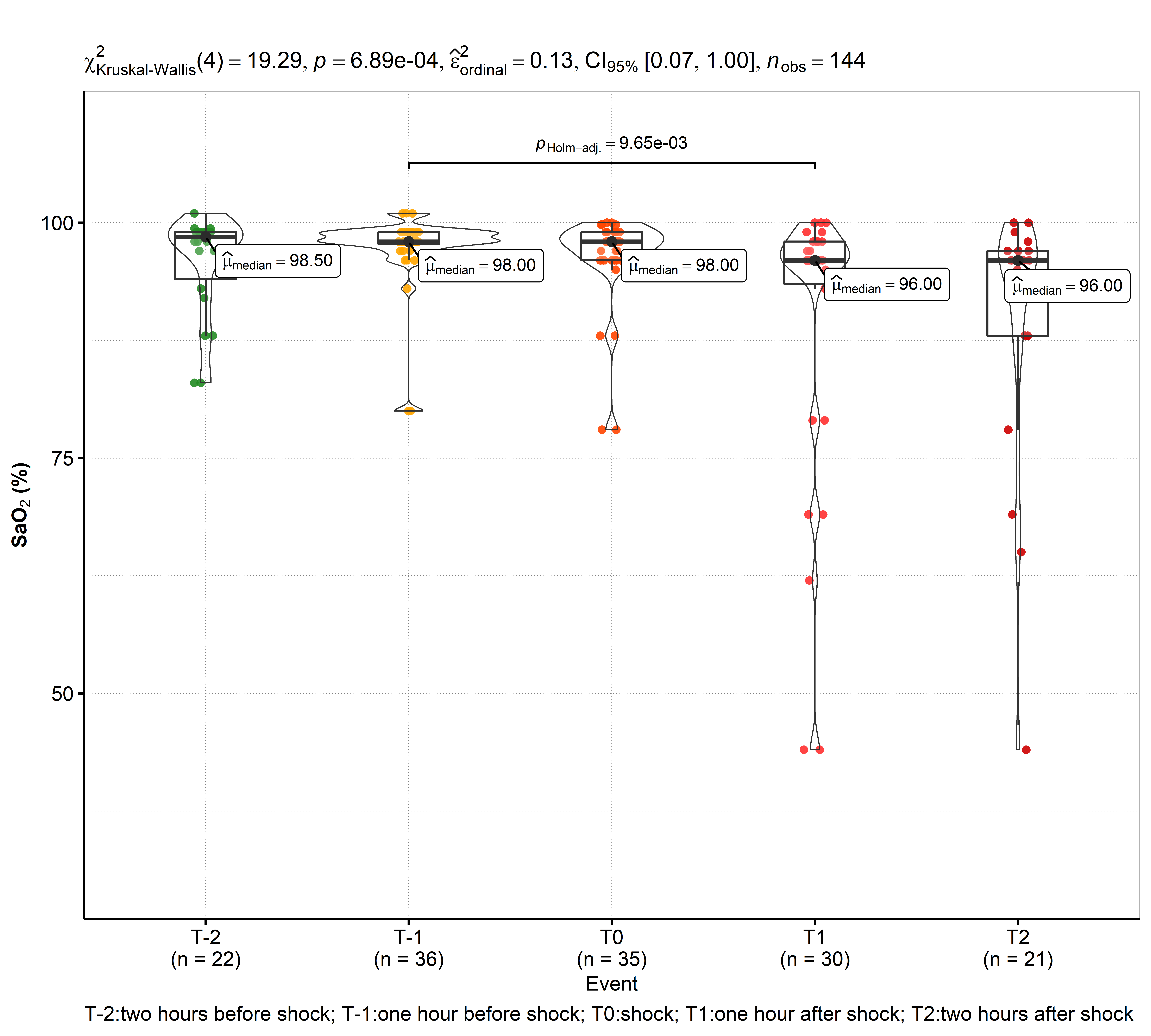  **Fig. S20**  Comparison of arterial oxygen saturation (SaO_2_) between the control group (CG) and the shock group (SG). |  | 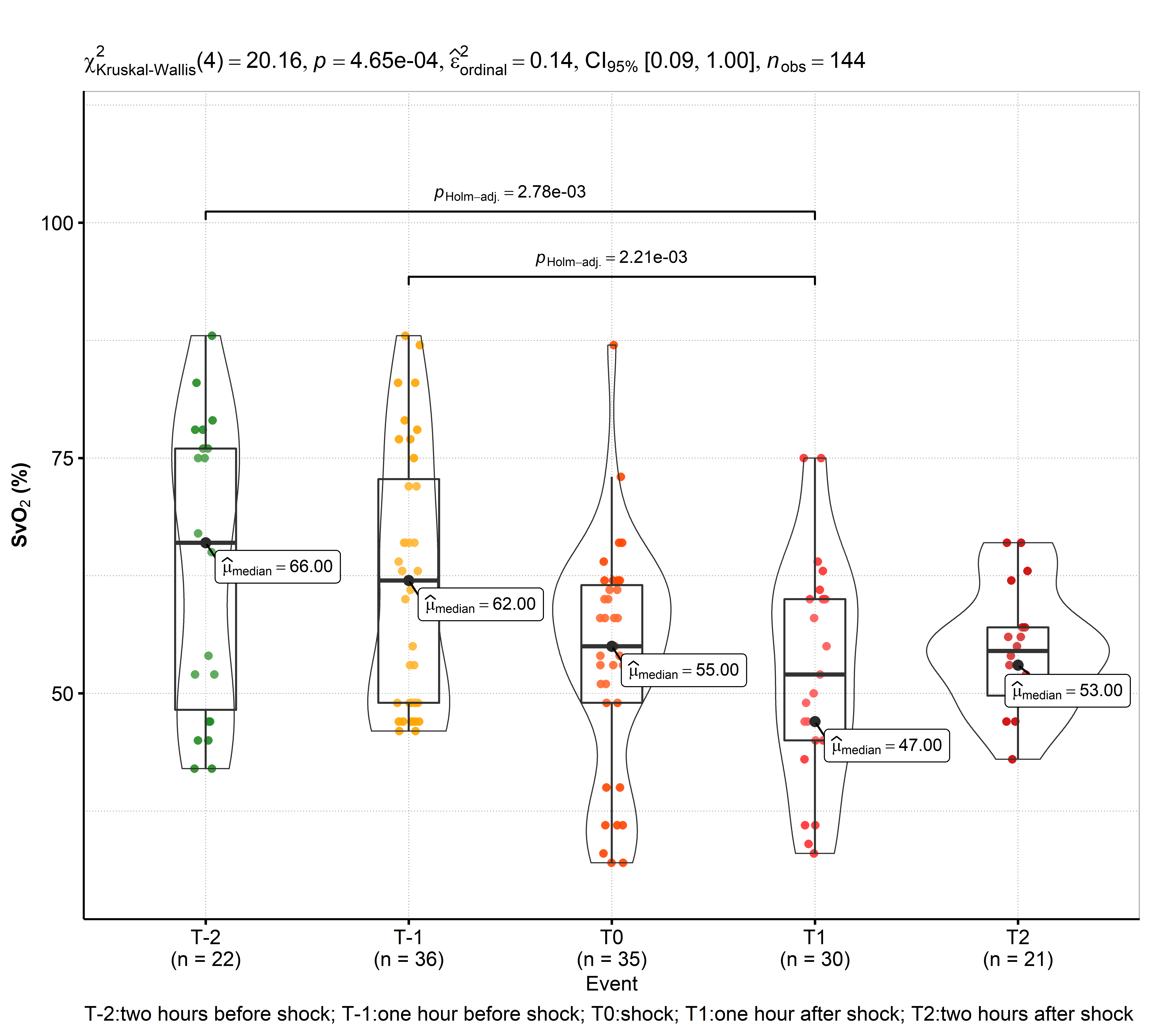  **Fig. S21**  Comparison of mixed venous saturation (SvO_2_) between the control group (CG) and the shock group (SG). |
| --- | --- | --- |

| 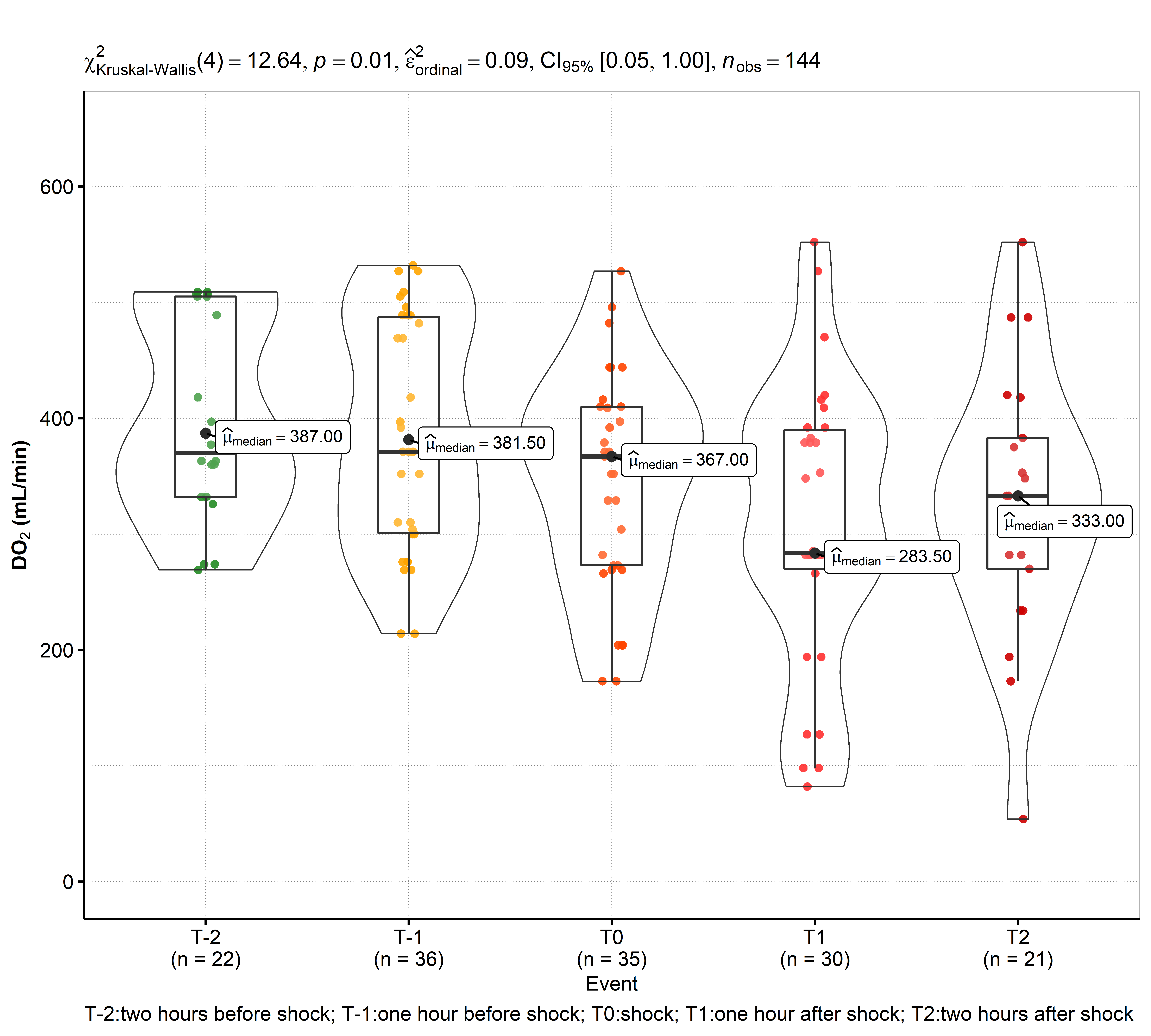  **Fig. S22**  Comparison of oxygen delivery (DO_2_) between the control group (CG) and the shock group (SG). |  | 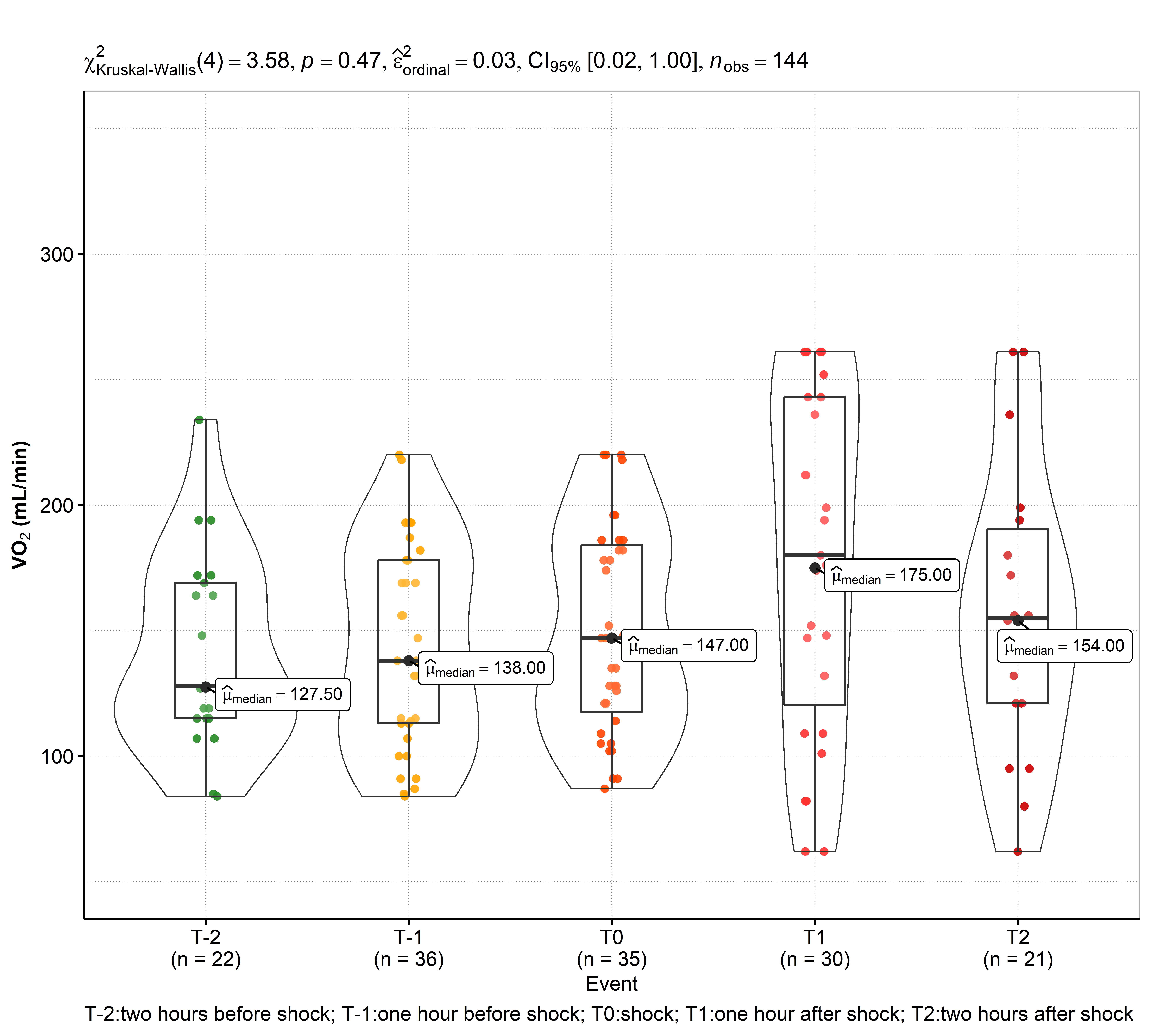  **Fig. S23**  Comparison of oxygen consumption (VO_2_) between the control group (CG) and the shock group (SG). |
| --- | --- | --- |

**
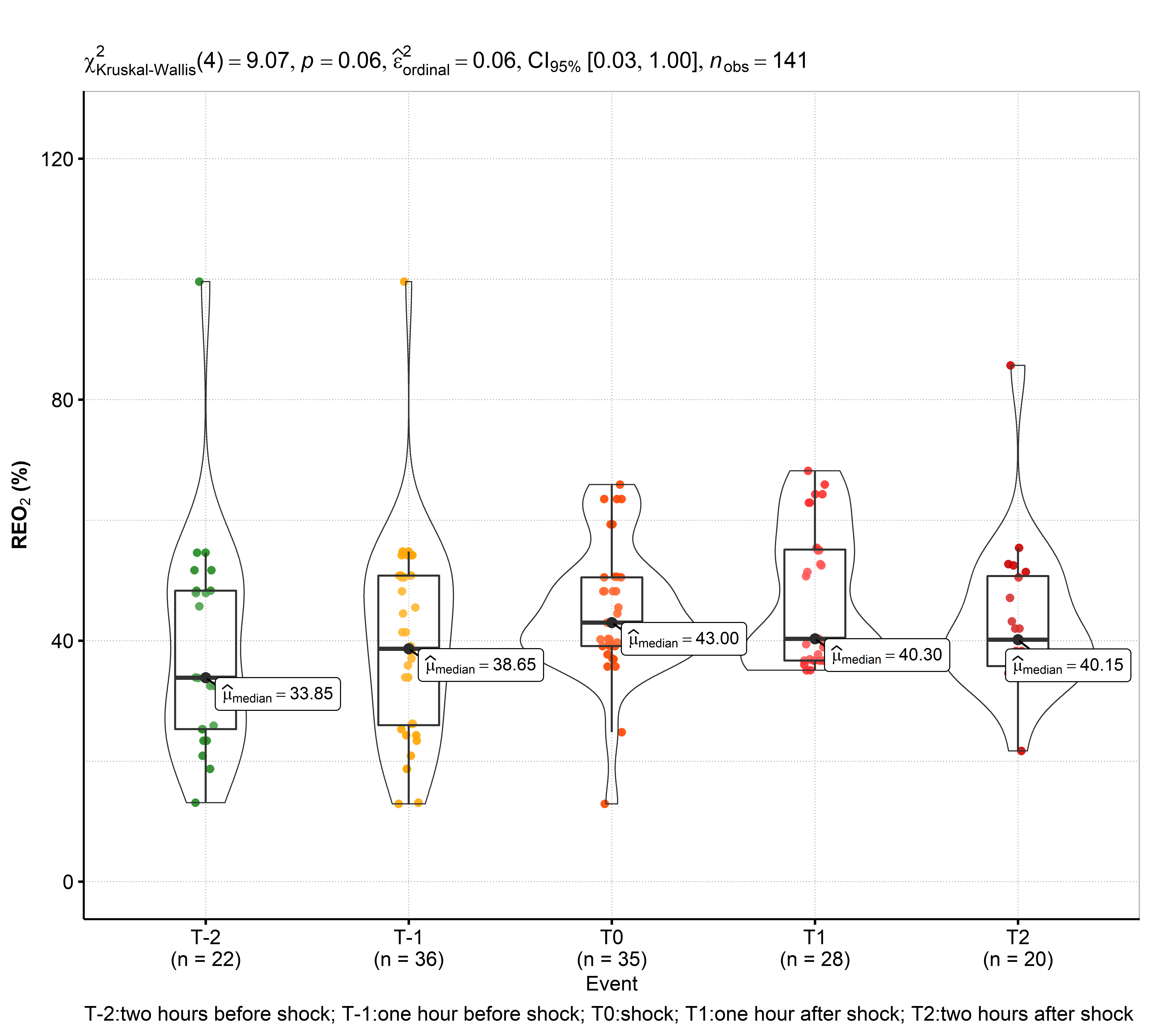
**

**Fig. S24**  Comparison of oxygen extraction ratio (REO_2_) between the control group (CG) and the shock group (SG).
