# Supplementary material for: Comparison of gastric reactance with commonly used perfusion markers in a swine hypovolemic shock model
Source: Intensive Care Med Exp. 2022 Nov 18;10:49. doi: 10.1186/s40635-022-00476-1 (PMC9674824; doi:10.1186/s40635-022-00476-1)
Supplement: Supplementary file 4 — Additional file 4: Table S3 Summary of hematology, blood chemistry test, electrolyte panel, renal and liver panel. Data presented as Median [IQR]; p for Kruskal–Wallis rank sum test. * For statistically significant p values < 0.05. ALP Alkaline phosphatase, ALT Aminotransferase alanine, AST Aminotransferase aspartate, CK Creatine kinase, K+ Potassium, LDH Lactate dehydrogenase, MCHC Mean corpuscular hemoglobin concentration, MCV Mean corpuscular volume, Na+ Sodium, PT Prothrombin time, PTT Partial thromboplastin time. [file 40635_2022_476_MOESM4_ESM.docx]

**Table S3** Summary of hematology, blood chemistry test, electrolyte panel, renal and liver panel.

| **Laboratory test** | **Control Group**  **N = 5** | **Pre-Shock**  **N = 16** | **Post-Shock**  **N = 16** | *p* |
| --- | --- | --- | --- | --- |
| Hematocrit (L/L) | 0.28 [0.3, 0.31] | 0.31 [0.3, 0.34] | 0.32 [0.3, 0.33] | 0.340 |
| Hemoglobin (g/dL) | 94.00 [91.0, 97.00] | 97.50 [83.8, 102.25] | 91.50 [84.2, 100.25] | 0.694 |
| Erythrocyte (x 10^6^/uL) | 5.30 [5.2, 5.80] | 5.50 [5.2, 6.00] | 5.30 [4.9, 5.90] | 0.879 |
| MCV (fL) | 55.00 [54.4, 55.50] | 57.00 [55.0, 58.00] | 55.00 [53.0, 56.00] | 0.212 |
| MCHC (g/dL) | 325.00 [303.0, 331.00] | 294.50 [282.2, 310.50] | 293.50 [287.8, 315.25] | 0.160 |
| Platelet (x 10^^3^/uL) | 300.00 [242.0, 408.00] | 366.00 [334.0, 468.00] | 368.00 [282.0, 414.00] | 0.403 |
| Total Solids (g/L) | 41.00 [38.5, 43.50] | 46.00 [43.0, 49.00] | 40.00 [38.0, 42.00] | 0.007* |
| Leukocytes (x 10^3^/uL) | 11.40 [11.4, 12.00] | 14.70 [13.4, 18.20] | 18.30 [14.1, 22.80] | 0.019* |
| Neutrophils (x 10^3^/uL) | 3.75 [2.8, 4.78] | 4.50 [3.7, 5.40] | 10.10 [7.8, 13.40] | <0.001* |
| Lymphocytes (x 10^3^/uL) | 5.80 [5.8, 7.20] | 9.30 [8.2, 12.50] | 6.80 [5.2, 8.00] | 0.004* |
| Monocytes (x 10^3^/uL) | 0.20 [0.1, 0.20] | 0.30 [0.1, 0.35] | 0.20 [0.1, 0.40] | 0.892 |
| Eosinophils (x 10^3^/uL) | 0.10 [0.1, 0.18] | 0.10 [0.0, 0.25] | 0.00 [0.0, 0.00] | 0.028* |
| Basophils (x 10^3^/uL) | 0.10 [0.1, 2.02] | 0.00 [0.0, 0.10] | 0.00 [0.0, 0.00] | 0.042* |
| Glucose (mmol/L) | 4.88 [4.1, 5.57] | 5.97 [5.0, 6.90] | 4.53 [3.7, 6.36] | 0.217 |
| Total_Bilirubin (umol/L) | 2.20 [1.2, 2.23] | 2.06 [1.1, 2.65] | 2.18 [1.6, 2.42] | 0.667 |
| AST (U/L) | 31.55 [26.8, 41.58] | 33.00 [28.2, 37.00] | 94.00 [66.0, 116.50] | <0.001* |
| ALT (U/L) | 38.00 [31.8, 43.65] | 34.00 [28.8, 39.50] | 32.00 [26.5, 41.50] | 0.695 |
| ALP (U/L) | 336.50 [231.2, 467.50] | 537.50 [476.0, 605.75] | 560.00 [504.5, 701.00] | 0.069 |
| CK (U/L) | 1,536.00 [1,412.0, 1,660.00] | 1,307.00 [1,080.2, 1,597.50] | 1,945.00 [1,336.5, 2,308.50] | 0.041* |
| LDH (U/L) | 524.50 [476.5, 581.75] | 866.50 [671.0, 1,073.25] | 769.00 [637.1, 943.00] | 0.068 |
| PT (s) | 10.70 [10.3, 16.30] | 16.00 [14.8, 16.88] | 14.80 [13.6, 16.45] | 0.450 |
| PTT (s) | 23.75 [23.5, 24.03] | 19.85 [16.0, 30.00] | 19.80 [14.2, 24.25] | 0.549 |

Data presented as Median [IQR]; *p* for Kruskal-Wallis rank sum test. * for statistically significant results *p* < 0.05.

*ALP* Alkaline phosphatase, *ALT* Aminotransferase alanine, *AST* Aminotransferase aspartate, *CK* Creatine kinase, *LDH* Lactate dehydrogenase, *MCHC* Mean corpuscular hemoglobin concentration, *MCV* Mean corpuscular volume, PT Prothrombin time, *PTT* Partial thromboplastin time.
