# Supplementary material for: Comparison of gastric reactance with commonly used perfusion markers in a swine hypovolemic shock model
Source: Intensive Care Med Exp. 2022 Nov 18;10:49. doi: 10.1186/s40635-022-00476-1 (PMC9674824; doi:10.1186/s40635-022-00476-1)
Supplement: Supplementary file 5 — Additional file 5: Table S4 XL p values for post hoc Dunn´s all pairs test for Kruskal–Wallis rank sum test. Data presented as Median [IQR]; CG Control Group, XL gastric reactance. Events by shock criterion (MAP ≤ 48 mmHg) are T-2: 2 h before shock; T-1: 1 h before shock; T0: shock; T1: 1 h after shock; T2: 2 h after shock. * For statistically significant p values < 0.05. Table S5 Lactate p values for post hoc Dunn´s all pairs test for Kruskal–Wallis rank sum test. Data presented as Median [IQR]; CG Control Group. Events by shock criterion (MAP ≤ 48 mmHg) are T-2: 2 h before shock; T-1: 1 h before shock; T0: shock; T1: 1 h after shock; T2: 2 h after shock. * For statistically significant p values < 0.05. Table S6 XL_Min p values for post hoc Durbin–Conover test for Friedman Test. Data presented as Median [IQR]; CG Control Group, XL_Min minimum XL value per subject per event. Events by shock criterion (MAP ≤ 48 mmHg) are T-2: 2 h before shock; T-1: 1 h before shock; T0: shock; T1: 1 h after shock; T2: 2 h after shock. * For statistically significant p values < 0.05. Table S7 Lac_Max p values for post hoc Durbin–Conover test for Friedman Test. Data presented as Median [IQR]; CG Control Group, Lac_Max maximum lactate value per subject per event. Events by shock criterion (MAP ≤ 48 mmHg) are T-2: 2 h before shock; T-1: 1 h before shock; T0: shock; T1: 1 h after shock; T2: 2 h after shock. * For statistically significant p values < 0.05. [file 40635_2022_476_MOESM5_ESM.docx]

**Table S4** XL p-values for post hoc Dunn´s all pairs test for Kruskal-Wallis rank sum test.

| **XL** Median  [Q1-Q3]  n | **CG**  8.93  [7.06-11.50]  n=57 | **T-2**  10.84  [8.18-14.68]  n=30 | **T-1**  14.89  [11.52-20.21]  n=39 | **T0**  15.60  [8.21-21.22]  n=37 | **T1**  19.78  [15.41-33.76]  n=36 | **T2**  25.70  [19.72-38.28]  n=21 |
| --- | --- | --- | --- | --- | --- | --- |
| **CG**  8.93  [7.06-11.50]  n=57 |  | 0.317 | <0.001* | 0.004* | <0.001* | <0.001* |
| **T-2**  10.84  [8.18-14.68]  n=30 | 0.317 |  | 0.317 | 0.375 | 0.003* | <0.001* |
| **T-1**  14.89  [11.52-20.21]  n=39 | <0.001* | 0.317 |  | 0.593 | 0.317 | 0.011* |
| **T0**  15.60  [8.21-21.22]  n=37 | 0.004* | 0.375 | 0.593 |  | 0.104 | 0.003* |
| **T1**  19.78  [15.41-33.76]  n=36 | <0.001* | 0.003* | 0.317 | 0.104 |  | 0.375 |
| **T2**  25.70  [19.72-38.28]  n=21 | <0.001* | <0.001* | 0.011* | 0.003* | 0.375 |  |

Data presented as Median [IQR]*; CG* Control Group, *XL* gastric reactance. Events by shock criterion (MAP ≤ 48 mmHg) are T-2: two hours before shock; T-1:one hour before shock; T0: shock; T1: one hour after shock; T2: two hours after shock. * for statistically significant results (*p*  < 0.05).

**Table S5** Lactate p-values for post hoc Dunn´s all pairs test for Kruskal-Wallis rank sum test.

| **Lactate**  Median  [Q1-Q3]  n | **CG**  2.10  [1.80 – 3.42]  n=60 | **T-2**  2.00  [1.75-2.80]  n=31 | **T-1**  2.30  [1.90-2.98]  n=34 | **T0**  2.90  [2.35-4.00]  n=35 | **T1**  5.75  [3.35-8.20]  n=34 | **T2**  8.90  [5.90-10.10]  n=21 |
| --- | --- | --- | --- | --- | --- | --- |
| **CG**  2.10  [1.80 – 3.42]  n=60 |  | 1.000 | 1.000 | 0.072 | <0.001* | <0.001* |
| **T-2**  2.00  [1.75-2.80]  n=31 | 1.000 |  | 1.000 | 0.031* | <0.001* | <0.001* |
| **T-1**  2.30  [1.90-2.98]  n=34 | 1.000 | 1.000 |  | 0.139 | <0.001* | <0.001* |
| **T0**  2.90  [2.35-4.00]  n=35 | 0.072 | 0.031* | 0.139 |  | 0.008* | <0.001* |
| **T1**  5.75  [3.35-8.20]  n=34 | <0.001* | <0.001* | <0.001* | 0.008* |  | 0.747 |
| **T2**  8.90  [5.90-10.10]  n=21 | <0.001* | <0.001* | <0.001* | <0.001* | 0.747 |  |

Data presented as Median [IQR]*; CG* Control Group. Events by shock criterion (MAP ≤ 48 mmHg) are T-2: two hours before shock; T-1:one hour before shock; T0: shock; T1: one hour after shock; T2: two hours after shock. * for statistically significant results (*p*  < 0.05).

**Table S6** XL_Min p-values for post hoc Durbin-Conover test for Friedman Test.

| **XL_Min** Median  [Q1-Q3]  n | **T-2**  8.45  [8.16-14.28]  n=11 | **T-1**  11.93  [9.65-14.23]  n=11 | **T0**  11.16  [6.69-17.40]  n=11 | **T1**  17.13  [14.58-26.14]  n=11 | **T2**  25.70  [20.36-37.91]  n=11 |
| --- | --- | --- | --- | --- | --- |
| **T-2**  8.45  [8.16-14.28]  n=11 |  | 1.000 | 1.000 | 0.016* | <0.001* |
| **T-1**  11.93  [9.65-14.23]  n=11 | 1.000 |  | 1.000 | 0.016* | <0.001* |
| **T0**  11.16  [6.69-17.40]  n=11 | 1.000 | 1.000 |  | 0.016* | <0.001* |
| **T1**  17.13  [14.58-26.14]  n=11 | 0.016* | 0.016* | 0.016* |  | 0.164 |
| **T2**  25.70  [20.36-37.91]  n=11 | <0.001* | <0.001* | <0.001* | 0.164 |  |

Data presented as Median [IQR]*; CG* Control Group, *XL_Min* minimum gastric reactance value per subject per event. Events by shock criterion (MAP ≤ 48 mmHg) are T-2: two hours before shock; T-1:one hour before shock; T0: shock; T1: one hour after shock; T2: two hours after shock. * for statistically significant results (*p*  < 0.05).

**Table S7** Lac_Max p-values for post hoc Durbin-Conover test for Friedman Test.

| **Lac_Max** Median  [Q1-Q3]  n | **T-2**  2.40  [2.20-3.00]  n=11 | **T-1**  2.50  [2.00-3.10]  n=11 | **T0**  2.70  [2.25-3.85]  n=11 | **T1**  6.20  [3.65-7.50]  n=11 | **T2**  9.50  [6.20-10.35]  n=11 |
| --- | --- | --- | --- | --- | --- |
| **T-2**  2.40  [2.20-3.00]  n=11 |  | 0.876 | 0.876 | <0.001* | <0.001* |
| **T-1**  2.50  [2.00-3.10]  n=11 | 0.876 |  | 0.476 | <0.001* | <0.001* |
| **T0**  2.70  [2.25-3.85]  n=11 | 0.876 | 0.476 |  | <0.001* | <0.001* |
| **T1**  6.20  [3.65-7.50]  n=11 | <0.001* | <0.001* | <0.001* |  | 0.006* |
| **T2**  9.50  [6.20-10.35]  n=11 | <0.001* | <0.001* | <0.001* | 0.006* |  |

Data presented as Median [IQR]*; CG* Control Group, *Lac_Max* maximum lactate value per subject per event. Events by shock criterion (MAP ≤ 48 mmHg) are T-2: two hours before shock; T-1:one hour before shock; T0: shock; T1: one hour after shock; T2: two hours after shock. * for statistically significant results (*p*  < 0.05).
