# Supplementary material for: Comparison of gastric reactance with commonly used perfusion markers in a swine hypovolemic shock model
Source: Intensive Care Med Exp. 2022 Nov 18;10:49. doi: 10.1186/s40635-022-00476-1 (PMC9674824; doi:10.1186/s40635-022-00476-1)
Supplement: Supplementary file 6 — Additional file 6: Table S8 Endoscopic and histological examinations of the fundus, the body, and the antrum of the stomach. Data presented as Median [IQR]; p for Kruskal–Wallis rank sum test. * For statistically significant results (p < 0.05). The macroscopic appearance of the mucosa was classified by a gastroenterologist following the empirical: scale 0—normal mucosa, 1—stippling or epithelial hemorrhage, 2—pale mucosa, 3—violet mucosa, and 4—marmoreal mucosa. Biopsies were qualitatively classified by a pathologist as "Inflammation & Edema" and "Superficial Detachment" following the empirical scale: 0%, 12.5%, 25%, 50%, 75%, and 100%. Events by shock criterion (MAP ≤ 48 mmHg) are T-2: 2 h before shock; T-1: 1 h before shock; T0: shock; T1: 1 h after shock; T2: 2 h after shock. Tables S9–S11 Control Group—endoscopic and histological examinations of the antrum, the body, and the fundus of subject C05. Tables S12–S14 Shock Group—endoscopic and histological examinations of the antrum, the body, and the fundus of subject C11. [file 40635_2022_476_MOESM6_ESM.docx]

**Table S8** Endoscopic and histological examinations of the fundus, the body, and the antrum of the stomach.

|  | **Control Group** | **Shock**  **Group** | | | | |  |
| --- | --- | --- | --- | --- | --- | --- | --- |
| ***Fundus*** | | | | | | | |
| **Variable** | **N = 10** | **T-2**  **N = 7** | **T-1**  **N = 4** | **T0**  **N = 9** | **T1**  **N = 11** | **T2**  **N = 9** | ***p*** |
| Inflammation-Edema (%) | 0.12  [0.1, 0.12] | 0.25  [0.1, 0.25] | 0.31  [0.1, 0.56] | 0.25  [0.1, 0.25] | 0.25  [0.2, 0.25] | 0.25  [0.1, 0.25] | 0.149 |
| Detachment (%) | 0.12  [0.1, 0.12] | 0.12  [0.1, 0.25] | 0.25  [0.2, 0.38] | 0.12  [0.1, 0.25] | 0.19  [0.1, 0.25] | 0.25  [0.1, 0.50] | 0.364 |
| Mucosa Class  (0-4 levels) | 0.00  [0.0, 0.00] | 0.00  [0.0, 1.00] | 2.50  [1.5, 3.25] | 2.00  [2.0, 2.00] | 2.00  [2.0, 2.00] | 3.00  [2.0, 3.00] | <0.001* |
| XL (-jΩ) | 8.76  [6.9, 10.88] | 8.45  [8.2, 11.63] | 18.04  [13.6, 23.98] | 16.43  [10.7, 23.16] | 25.67  [10.7, 31.06] | 31.50  [20.3, 39.85] | 0.020* |
| ***Body*** | | | | | | | |
| **Variable** | **N = 10** | **T-2**  **N = 7** | **T-1**  **N = 4** | **T0**  **N = 8** | **T1**  **N = 12** | **T2**  **N = 9** | ***p*** |
| Inflammation-Edema (%) | 0.12  [0.1, 0.22] | 0.25  [0.2, 0.50] | 0.25  [0.2, 0.38] | 0.12  [0.1, 0.16] | 0.25  [0.1, 0.44] | 0.25  [0.2, 0.25] | 0.021* |
| Detachment (%) | 0.12  [0.1, 0.22] | 0.12  [0.1, 0.19] | 0.25  [0.2, 0.25] | 0.12  [0.1, 0.25] | 0.12  [0.1, 0.19] | 0.50  [0.1, 0.50] | 0.132 |
| Mucosa Class  (0-4 levels) | 0.00  [0.0, 0.00] | 0.00  [0.0, 1.00] | 2.50  [1.5, 3.25] | 2.00 [2.0, 2.00] | 2.00  [2.0, 2.25] | 3.00  [3.0, 3.00] | <0.001* |
| XL (-jΩ) | 8.76  [6.9, 10.88] | 8.45  [8.2, 11.63] | 18.04  [13.6, 23.98] | 15.18  [9.8, 18.82] | 26.83  [13.2, 34.66] | 31.50  [22.9, 38.28] | 0.011* |
| ***Antrum*** | | | | | | | |
| **Variable** | **N = 10** | **T-2**  **N = 7** | **T-1**  **N = 4** | **T0**  **N = 8** | **T1**  **N = 11** | **T2**  **N = 10** | ***p*** |
| Inflammation-Edema (%) | 0.12  [0.1, 0.25] | 0.50  [0.2, 0.50] | 0.25  [0.2, 0.31] | 0.19  [0.1, 0.25] | 0.25  [0.2, 0.50] | 0.19  [0.1, 0.25] | 0.043* |
| Detachment (%) | 0.12  [0.1, 0.12] | 0.12  [0.0, 0.12] | 0.12  [0.1, 0.12] | 0.12  [0.1, 0.12] | 0.12  [0.1, 0.25] | 0.12  [0.1, 0.22] | 0.096 |
| Mucosa Class  (0-4 levels) | 0.00  [0.0, 0.00] | 0.00  [0.0, 1.00] | 2.50  [1.5, 3.25] | 2.00  [2.0, 2.00] | 2.00  [2.0, 2.50] | 2.50  [2.0, 3.00] | <0.001* |
| XL (-jΩ) | 8.45  [6.8, 10.88] | 8.45  [8.2, 11.63] | 18.04  [13.6, 23.98] | 16.34  [9.8, 20.34] | 27.06  [11.5, 35.56] | 28.60  [20.5, 38.00] | 0.010* |

Data presented as Median [IQR]; p for Kruskal-Wallis rank sum test. *for statistically significant results (*p* < 0.05). The macroscopic appearance of the mucosa was classified by a gastroenterologist following the empirical scale: 0-normal mucosa, 1-stippling or epithelial hemorrhage, 2-pale mucosa, 3-violet mucosa, and 4-marmoreal mucosa. Biopsies were qualitatively classified by a pathologist as "Inflammation & Edema" and "Superficial Detachment" following the empirical scale: 0%, 12.5%, 25%, 50%, 75%, and 100%. Events by shock criterion (MAP ≤ 48 mmHg) are T-2: two hours before shock; T-1:one hour before shock; T0: shock; T1: one hour after shock; T2: two hours after shock.

**Table S9** Control Group - Endoscopic and histological examinations of the antrum of subject C05.

| **Variable** | **Initial** | **Final** |
| --- | --- | --- |
| **Antrum**  Histopathologic image 100x | 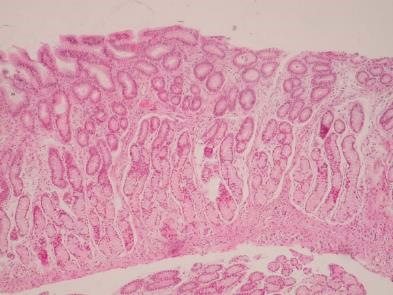 | 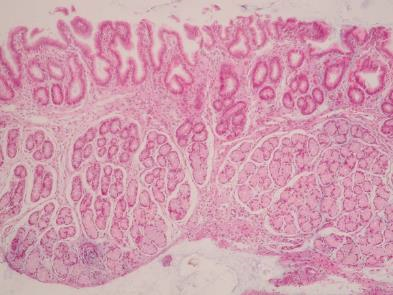 |
| Inflammation-Edema (%) | 12.50% | 12.50% |
| Detachment (%) | 12.50% | 12.50% |
| Mucosa Class  (0-4 levels) | 0 – normal mucosa | 0 – normal mucosa |
| Lactate (mmol/L) | 2.0 | 1.8 |
| XL (-jΩ) | 9.04 | 8.59 |

The macroscopic appearance of the mucosa was classified by a gastroenterologist following the empirical scale: 0-normal mucosa, 1-stippling or epithelial hemorrhage, 2-pale mucosa, 3-violet mucosa, and 4-marmoreal mucosa. Biopsies were qualitatively classified by a pathologist as "Inflammation & Edema" and "Superficial Detachment" following the empirical scale: 0%, 12.5%, 25%, 50%, 75%, and 100%. Control group biopsy samples by protocol procedures: Initial - basal condition, Final - after 5 hours without hemorrhage.

**Table S10** Control Group **-** Endoscopic and histological examinations of the body of subject C05.

| **Variable** | **Initial** | **Final** |
| --- | --- | --- |
| **Body**  Histopathologic image 100x | 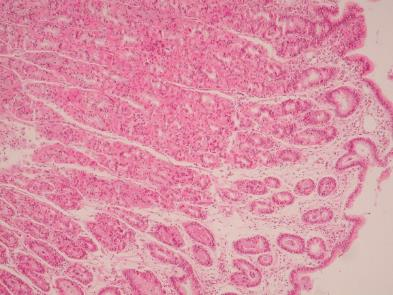 | 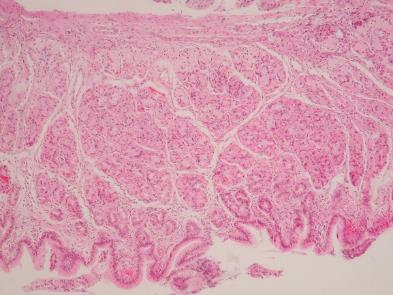 |
| Inflammation-Edema (%) | 25.00% | 12.50% |
| Detachment (%) | 12.50% | 25.00% |
| Mucosa Class  (0-4 levels) | 0 – normal mucosa | 0 – normal mucosa |
| Lactate (mmol/L) | 2.0 | 1.8 |
| XL (-jΩ) | 9.04 | 8.59 |

The macroscopic appearance of the mucosa was classified by a gastroenterologist following the empirical scale: 0-normal mucosa, 1-stippling or epithelial hemorrhage, 2-pale mucosa, 3-violet mucosa, and 4-marmoreal mucosa. Biopsies were qualitatively classified by a pathologist as "Inflammation & Edema" and "Superficial Detachment" following the empirical scale: 0%, 12.5%, 25%, 50%, 75%, and 100%. Control group biopsy samples by protocol procedures: Initial - basal condition, Final - after 5 hours without hemorrhage.

**Table S11** Control Group **-** Endoscopic and histological examinations of the fundus of subject C05.

| **Variable** | **Initial** | **Final** |
| --- | --- | --- |
| **Fundus**  Histopathologic image 100x | 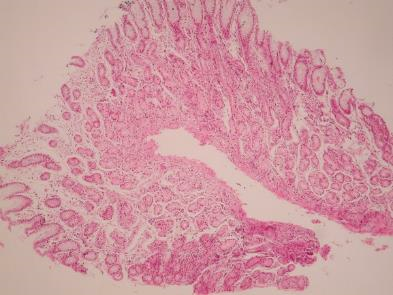 | 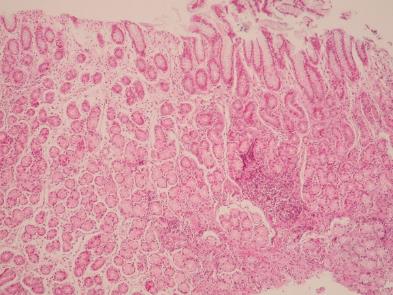 |
| Inflammation-Edema (%) | 12.50% | 12.50% |
| Detachment (%) | 12.50% | 12.50% |
| Mucosa Class  (0-4 levels) | 0 – normal mucosa | 0 – normal mucosa |
| Lactate (mmol/L) | 2.0 | 1.8 |
| XL (-jΩ) | 9.04 | 8.59 |

The macroscopic appearance of the mucosa was classified by a gastroenterologist following the empirical scale: 0-normal mucosa, 1-stippling or epithelial hemorrhage, 2-pale mucosa, 3-violet mucosa, and 4-marmoreal mucosa. Biopsies were qualitatively classified by a pathologist as "Inflammation & Edema" and "Superficial Detachment" following the empirical scale: 0%, 12.5%, 25%, 50%, 75%, and 100%. Control group biopsy samples by protocol procedures: Initial - basal condition, Final - after 5 hours without hemorrhage.

**Table S12** Shock Group **-** Endoscopic and histological examinations of the antrum of subject C11.

| **Variable** | **Initial** | **T-1** | **T0** | **T2** |
| --- | --- | --- | --- | --- |
| **Antrum**  Histopathologic image 100x | 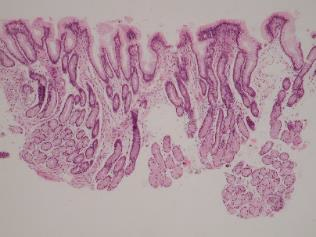 | 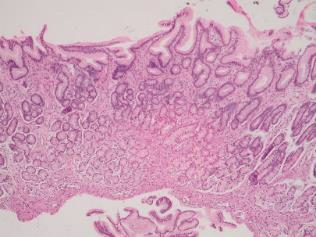 | 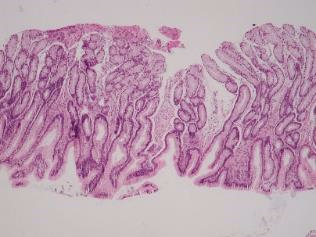 | 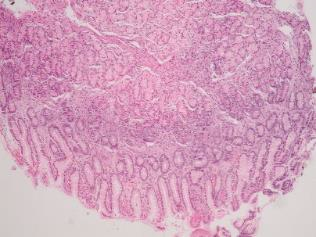 |
| **Antrum**  Histopathologic image 400x | - | - | - | 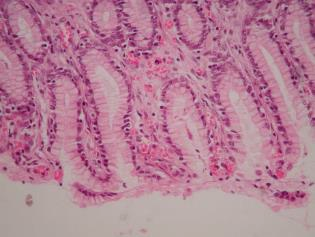 |
| Inflammation-Edema (%) | 12.50% | 12.50% | 12.50% | 12.50% |
| Detachment (%) | 12.50% | 25.00% | 12.50% | 50.00% |
| Mucosa Class   - 1. levels) | 0 – normal mucosa | 0 – normal mucosa | 2 – pale mucosa | 3 – violet mucosa |
| Lactate (mmol/L) | 3.7 | 2.4 | 5.2 | 13.5 |
| XL (-jΩ) | 12.07 | 13.56 | 24.19 | 37.17 |

The macroscopic appearance of the mucosa was classified by a gastroenterologist following the empirical scale: 0-normal mucosa, 1-stippling or epithelial hemorrhage, 2-pale mucosa, 3-violet mucosa, and 4-marmoreal mucosa. Biopsies were qualitatively classified by a pathologist as "Inflammation & Edema" and "Superficial Detachment" following the empirical scale: 0%, 12.5%, 25%, 50%, 75%, and 100%. by shock criterion (MAP ≤ 48 mmHg) Shock group biopsy samples by protocol procedures: Initial - basal condition; T-1 - one hour before shock; T0 - shock; T2- two hours after shock.

**Table S13** Shock Group **-** Endoscopic and histological examinations of the body of subject C11.

| **Variable** | **Initial** | **T-1** | **T0** | **T2** |
| --- | --- | --- | --- | --- |
| **Body**  Histopathologic image 100x | 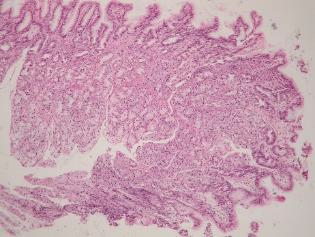 | 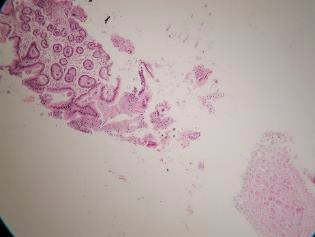 | 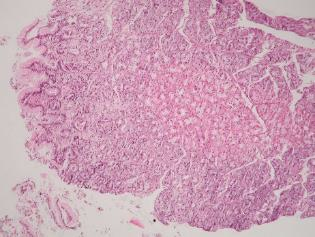 | 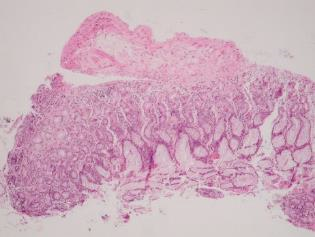 |
| **Body**  Histopathologic image 400x | - | - | - | 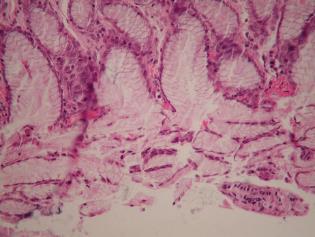 |
| Inflammation-Edema (%) | 12.50% | 12.50% | 12.50% | 12.50% |
| Detachment (%) | 12.50% | 25.00% | 25.00% | 50.00% |
| Mucosa Class   - 1. levels) | 0 – normal mucosa | 0 – normal mucosa | 2 – pale mucosa | 3 – violet mucosa |
| Lactate (mmol/L) | 3.7 | 2.4 | 5.2 | 13.5 |
| XL (-jΩ) | 12.07 | 13.56 | 24.19 | 37.17 |

The macroscopic appearance of the mucosa was classified by a gastroenterologist following the empirical scale: 0-normal mucosa, 1-stippling or epithelial hemorrhage, 2-pale mucosa, 3-violet mucosa, and 4-marmoreal mucosa. Biopsies were qualitatively classified by a pathologist as "Inflammation & Edema" and "Superficial Detachment" following the empirical scale: 0%, 12.5%, 25%, 50%, 75%, and 100%. Events by shock criterion (MAP ≤ 48 mmHg) Shock group biopsy samples by protocol procedures: Initial - basal condition; T-1 - one hour before shock; T0 - shock; T2- two hours after shock.

**Table S14** Shock Group **-** Endoscopic and histological examinations of the fundus of subject C11.

| **Variable** | **Initial** | **T-1** | **T0** | **T2** |
| --- | --- | --- | --- | --- |
| **Fundus**  Histopathologic image 100x | 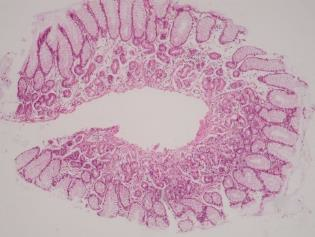 | 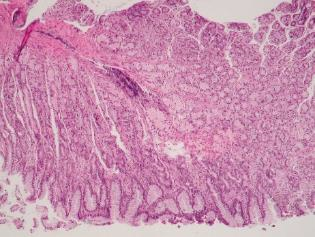 | 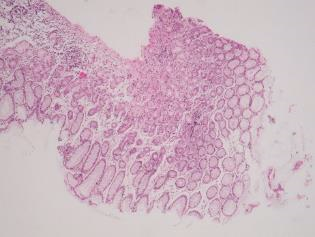 | 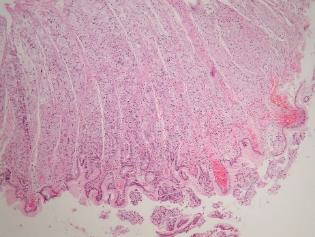 |
| **Fundus**  Histopathologic image 400x | - | - | - | 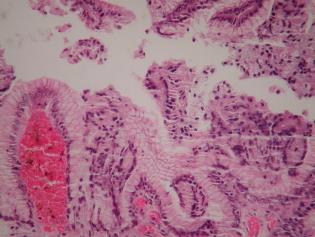 |
| Inflammation-Edema (%) | 12.50% | 12.50% | 12.50% | 12.50% |
| Detachment (%) | 12.50% | 25.00% | 12.50% | 50.00% |
| Mucosa Class   - 1. levels) | 0 – normal mucosa | 0 – normal mucosa | 2 – pale mucosa | 3 – violet mucosa |
| Lactate (mmol/L) | 3.7 | 2.4 | 5.2 | 13.5 |
| XL (-jΩ) | 12.07 | 13.56 | 24.19 | 37.17 |

The macroscopic appearance of the mucosa was classified by a gastroenterologist following the empirical scale: 0-normal mucosa, 1-stippling or epithelial hemorrhage, 2-pale mucosa, 3-violet mucosa, and 4-marmoreal mucosa. Biopsies were qualitatively classified by a pathologist as "Inflammation & Edema" and "Superficial Detachment" following the empirical scale: 0%, 12.5%, 25%, 50%, 75%, and 100%. Events by shock criterion (MAP ≤ 48 mmHg) Shock group biopsy samples by protocol procedures: Initial - basal condition; T-1 - one hour before shock; T0 - shock; T2- two hours after shock.
